# The line is drawn, the fate is cast: urban–rural inequalities in the timing of initial health check-ups in China

**DOI:** 10.3389/fsoc.2026.1702900

**Published:** 2026-05-08

**Authors:** Siyuan Chen, Piet Bracke, Katrijn Delaruelle

**Affiliations:** Department of Sociology, Ghent University, Ghent, Belgium

**Keywords:** China, diffusion of innovation, fundamental cause theory, health check-ups, *hukou*

## Abstract

**Introduction:**

Despite sustained advances in medical innovation and expanded access to healthcare services, inequalities in the uptake of health check-ups persist, pointing to enduring mechanisms in the reproduction of health inequalities. Although Fundamental Cause Theory (FCT) has been widely used to examine such inequalities, it has largely been operationalized at the individual level, with limited attention to institutional arrangements and insufficient consideration of how mechanisms of inequality shift over time.

**Methods:**

Drawing on the 2014 life history data from the China Health and Retirement Longitudinal Study (CHARLS), this research employs accelerated failure time (AFT) models to assess how China’s *hukou* system operates as an institutional determinant shaping inequalities in the timing of initial health check-ups. We analyze blood pressure tests and blood tests for glucose or cholesterol, vision and dental exams, and breast and prostate cancer screenings to assess how inequality unfolds across different stages of medical innovation diffusion.

**Results:**

The results indicate that urban *hukou* holders consistently undergo health check-ups earlier than their rural counterparts across all types of services. Among those with rural *hukou*, urban residence is associated with earlier uptake, particularly for blood tests, dental exams, and vision exams. As medical innovations diffuse, *hukou*-based inequalities tend to narrow for services at later stages of diffusion but widen for those at earlier stages.

**Discussion:**

This research reveals a dynamic substitution of mechanisms through which health inequalities are reproduced, highlighting the enduring institutional influence of the *hukou* system and its interaction with the diffusion of medical innovations in sustaining health inequalities over time.

## 1 Introduction

Health check-ups are a widely adopted preventive measure that facilitates early disease detection and reduces health risks. Despite sustained efforts to improve service accessibility, utilization remains uneven across sociodemographic groups. Individuals with lower socioeconomic status (SES) are less likely to use these services and more likely to delay uptake beyond recommended timeframes (Gong et al., 2016; De Prez et al., 2021; Xia et al., 2023). This raises a central sociological question: why do health inequalities persist despite the expansion of health resources? Fundamental Cause Theory (FCT) provides a theoretical framework for addressing this question. According to FCT, individuals with greater socioeconomic advantages are better able to leverage flexible resources, such as wealth, power, and prestige, to access health-protective opportunities, thereby reducing morbidity and mortality (Link and Phelan, 1995). This pattern persists across time, place, and health domains, even as disease profiles evolve and medical innovations advance, because when one mechanism underlying health inequalities weakens, another emerges to reproduce or even intensify these inequalities (Link et al., 1998; Zapata-Moya et al., 2023). Building on this framework, a substantial body of empirical research over the past three decades has examined FCT through three main approaches (Clouston and Link, 2021). The *disease preventability* approach compares socioeconomic gradients in morbidity and mortality across health outcomes with varying preventability (Rydland et al., 2020), the *changes in preventability* approach examines how inequalities shift before and after preventive advancements (Chang and Lauderdale, 2009; Zapata-Moya et al., 2019), and the *manipulated preventability* approach evaluates whether health inequalities emerge under the influence of randomly assigned interventions that require flexible resources for optimal uptake to extract maximum benefit (Yang et al., 2014). While these approaches offer substantial empirical support for FCT, they have been critiqued for insufficiently addressing the context-specific and dynamic nature of the social processes that (re)produce health inequalities (Beckfield et al., 2013; Zapata-Moya et al., 2023).

FCT originated in the US, where the liberal welfare state model emphasizes market-driven allocation of health resources. Such a system tends to amplify the influence of individual SES on health care access (Beckfield and Krieger, 2009; Beckfield et al., 2015). However, emerging evidence from other welfare state regimes suggests that the applicability of FCT is contingent upon institutional and policy contexts (Beckfield et al., 2013; Bakhtiari et al., 2018; De Prez et al., 2021). In such contexts, institutional interventions such as universal health coverage or the organized preventive screening programs can mitigate health inequalities by weakening the association between individual SES and access to health-protective resources (Olafsdottir, 2007; Clouston and Link, 2021; De Prez et al., 2022). For example, in countries with organized cancer screening programs, inequalities in participation in emerging preventive services tend to be less pronounced than in those relying on opportunistic screening strategies, as the institutional design of screening constrains the mechanisms through which inequalities are produced (Willems and Bracke, 2018; Jolidon et al., 2020; De Prez et al., 2021). Conversely, when institutions discriminate against low-SES groups and unequally allocate life chances and health resources, health inequalities are likely to be exacerbated (Colen et al., 2018; Barnes et al., 2023). Nevertheless, whether and to what extent the mechanisms proposed by FCT operate consistently across different healthcare systems and policy environments, particularly in non-Western contexts with distinct institutional arrangements, remains underexplored. Addressing this gap is essential for assessing the broader applicability of FCT across diverse sociopolitical settings, with varying structural forces shaping the distribution of health resources.

Furthermore, besides its limited attention to the role of institutional factors in shaping health inequalities, prior empirical research has insufficiently addressed the underlying meta-hypothesis regarding the substitution of mechanisms that mediate the relationship between social determinants and health outcomes (Zapata-Moya et al., 2023). As postulated by FCT, shifts in disease profiles and preventive innovations give rise to new mechanisms that supplant those in decline, thereby sustaining the association between social position and health. Although a few studies have begun to integrate FCT with Diffusion of Innovation (DOI) theory to examine the dynamic process of mechanism substitution (Zapata-Moya et al., 2019, 2023; Weiss et al., 2020; Rydland et al., 2022), such efforts remain scarce and highly context-specific, with limited exploration across broader institutional settings. The substitution of mechanisms is neither random nor exogenous but embedded within institutional contexts. Institutional arrangements can amplify or attenuate the operation of the mechanisms identified by FCT. Therefore, further cross-national research is also needed to clarify how institutional factors condition the dynamic reproduction of health inequalities.

To address these limitations, this study introduces China’s household registration (*hukou*) system as a fundamental institutional determinant to investigate how institutional arrangements contribute to the persistence and reproduction of inequalities in the timing of health check-ups amid ongoing healthcare expansion in a non-Western context. Specifically, we aim to address two research questions: (1) Does the *hukou* system shape inequalities in the timing of initial health check-ups between urban and rural *hukou* holders? and (2) How do these inequalities evolve as health resources become more widely available?

## 2 Background and hypotheses

### 2.1 *Hukou* as a fundamental cause in the initiation of health check-ups

This study conceptualizes China’s *hukou* system as a fundamental cause of health inequalities, thereby contextualizing FCT to a non-Western setting. Established in the early 1950s, the *hukou* system was initially designed to divide the population into urban or rural *hukou* identity to restrict rural-to-urban migration and determine eligibility for state-allocated resources and entitlements, thereby serving the needs of early socialist industrialization centered in urban areas in China (Cheng and Selden, 1994; Wu and Treiman, 2004; Song and Smith, 2021). Following reforms in the late 1970s, restrictions on rural-to-urban migration were gradually relaxed to meet urban labor demands, resulting in a great migration, with the number of migrant workers in cities reaching approximately 221 million by 2010 (Chan and Zhang, 1999; Zhu, 2007; Liang, 2016; Chen et al., 2024). Although many migrant workers have settled in urban areas, most continue to face barriers to obtaining an urban *hukou* and are therefore institutionally excluded from accessing social benefits closely tied to *hukou* status (Li et al., 2015; Song and Smith, 2021). In general, since its establishment, the *hukou* system has functioned as a central institutional arrangement shaping the unequal distribution of flexible resources emphasized by FCT, including economic capital, health literacy, social welfare, medical insurance, and geographic proximity to healthcare. These intertwined processes embedded in the *hukou* system underpin persistent health inequalities.

One of the key mechanisms by which the *hukou* system produces and sustains health inequalities lies in shaping individuals’ SES (Liu, 2005). As one of the most fundamental institutional markers of social stratification in China, the *hukou* system allocates differential life chances, consistently privileging urban *hukou* holders while systematically disadvantaging their rural counterparts across multiple domains (Wu and Treiman, 2004; Xiao and Bian, 2018; Wu, 2019). Previous research has consistently documented that, compared to their rural counterparts, urban *hukou* holders enjoy significant advantages in educational attainment (Wu, 2011), which translates into higher health literacy, greater self-efficacy in managing health conditions (Ross and Wu, 1995; Delaruelle et al., 2015), and a higher likelihood of utilizing regular health check-ups. In terms of occupation, prior to the relaxation of *hukou*-based migration restrictions, rural *hukou* holders were largely confined to agricultural labor. Following the policy relaxation, some of them remained in agriculture, while others migrated to urban areas. However, most rural-to-urban migrants found employment in low-skilled, physically demanding sectors such as construction and manufacturing, where jobs were often unstable, poorly paid, and offered limited prospects for upward mobility (Wang et al., 2015; Zhang and Wu, 2017; Ma, 2018; Stainback and Tang, 2019). By contrast, urban *hukou* holders have greater opportunities to employment in the public sector or state-owned enterprises (Li et al., 2015), which tend to offer more stable and better-compensated positions. As a result, rural *hukou* holders accumulate less economic capital, experience greater financial insecurity, and consequently have reduced access to regular health check-ups due to affordability constraints.

Moreover, beyond its role in shaping socioeconomic inequalities, the *hukou* system also reinforces health inequalities by unequally distributing other flexible resources, such as social welfare benefits (Wu, 2019), influencing access to preventive health services. Urban *hukou* holders, who constitute the majority of employees in state-owned enterprises and public sectors, enjoy privileged access to a range of welfare provisions, including subsidized housing, employer-sponsored health check-ups, and comprehensive pension schemes (Logan et al., 2009; Afridi et al., 2015). These institutional advantages enhance their capacity and opportunity to engage in timely preventive care. In contrast, rural *hukou* holders, whether residing in villages or migrating to cities, are excluded from the urban welfare system (Zhu and Walker, 2018; Wu, 2019) and lack comparable institutional support. Migrant workers in urban areas are structurally disadvantaged in employer type, being disproportionately employed in private-sector firms rather than government or state-affiliated institutions (Zhang and Wu, 2017; Hung, 2022), often under short-term or informal contracts that offer minimal access to social insurance or pension schemes (Zhu and Walker, 2018), which further entrenches health inequalities.

Additionally, the *hukou* system reinforces inequalities in health check-up utilization through the segmentation of medical insurance schemes. These schemes, shaped by *hukou*-based distinctions, contribute to uneven insurance coverage and place certain groups at economic disadvantage when seeking care (Ma et al., 2020). Urban *hukou* holders are more likely to be employed in the public sector and formal urban industries, which provide access to Urban-Employee-Based Basic Medical Insurance (UEBMI) with higher reimbursement rates and broader coverage (Yang et al., 2020; Dong et al., 2021). In contrast, rural *hukou* holders, whether engaged in agricultural work in rural areas or employed informally in cities, remain excluded from comparable insurance arrangements. As a result, most are covered by the New Cooperative Medical System (NCMS), which provides lower reimbursement rates and limited disease coverage (Lei and Lin, 2009; Zhu and Österle, 2017; Dong et al., 2021). These arrangements create structural barriers to equitable preventive care and further reproduce health inequalities along *hukou* lines.

Furthermore, residential disparities intertwined with the *hukou* system represent an additional factor reinforcing inequalities in health check-up utilization. Before the relaxation of strict rural-to-urban migration regulations, rural *hukou* holders were largely confined to rural areas with poorer medical infrastructure and mainly relied on “barefoot doctors” for health consultation and treatment (Zhang and Unschuld, 2008), effectively restricting their access to the higher-quality healthcare resources concentrated in urban regions (Anand et al., 2008). Following the reform that enabled greater population mobility, many rural *hukou* holders migrated to cities and benefited from the spillover effects of urban residence, such as employment opportunities in urban industries, which enhanced their financial capacity to afford healthcare. Additionally, they gained access to improved health-related social capital, which encouraged prioritization of preventive check-ups (Palmer and Xu, 2013). Combined with closer geographic proximity to high-quality medical facilities, these factors collectively contributed to increased utilization of health check-ups among rural *hukou* holders living in cities (Zheng et al., 2018). However, such improvements do not translate into equal ability to deploy the health-promoting resources compared to their urban *hukou* counterparts. As discussed above, rural *hukou* holders, despite relocating to urban areas, continue to face multiple disadvantages, including financial barriers, health literacy, welfare entitlements, and insurance coverage. These ongoing challenges reduce their ability to access high-quality services. Hence, geographic inequalities in the distribution of medical resources intersect with institutional arrangements rooted in the *hukou* system to further perpetuate inequalities in health check-up utilizations.

Overall, as an institutional arrangement, the *hukou* system fundamentally shapes individuals’ access to multiple flexible resources emphasized by FCT, including SES, social welfare, medical insurance, and geographic proximity to medical facilities, thereby perpetuating and reproducing structural inequalities that consistently privilege urban *hukou* holders. Based on the above theoretical reasoning, we propose the following hypotheses:

*Hypothesis 1: Urban hukou holders initiate health check-ups earlier than rural hukou holders, regardless of check-up type.*

*Hypothesis 2: Among rural hukou holders, urban residents initiate health check-ups earlier than rural residents, regardless of check-up type.*

### 2.2 Persistent health inequalities: intersections with diffusion of innovation theory

As previously discussed, the persistence of health inequalities reflects the meta-hypothesis that new mechanisms can replace earlier ones in (re)producing health inequalities as disease profiles shift and medical technologies advance (Freese and Lutfey, 2011). This dynamic process sustains the persistent relationships between social determinants and health outcomes. Yet, while FCT posits that flexible resources enable advantaged groups to maintain health advantages across times, places, and health domains, it does not provide a framework that can be readily operationalized in empirical research to test this meta-hypothesis.

Integrating FCT with the DOI theory helps to address this gap. By introducing an S-shaped diffusion curve to capture the diffusion of medical innovations at the population level and linking different stages of diffusion to hypothesized patterns of inequality, this approach transforms the theoretical argument of mechanism substitution into an empirically testable question. Rather than addressing solely whether health inequalities exist at a static point in time, it enables researchers to assess how inequalities evolve across multiple medical innovations at different stages of diffusion, thereby providing a clear empirical strategy for identifying how new mechanisms emerge to replace older ones that produce health inequalities.

Originally proposed and elaborated by Everett Rogers, the DOI theory posits that innovations spread through populations in an S-shaped curve, influenced by both social context and individual traits (Zapata-Moya et al., 2019). The diffusion process unfolds in five stages, including innovators, early adopters, early majority, late majority, and laggards, based on the cumulative adoption rate at the population level. Structural and individual characteristics shape not only who benefits from them but also when innovations are adopted. As diffusion of medical innovations progresses, corresponding patterns of health inequalities emerge and evolve. Zapata-Moya et al. (2019) illustrates the hypothetical pattern of diffusion of medical innovations and related health inequalities.

Individuals in higher social positions tend to access and adopt new medical innovations earlier, leading to health inequalities emerging and increasing during the early stages of diffusion, such as innovators and early adopters stages. At these early stages, new medical innovations and health screening programs are typically characterized by higher out-of-pocket costs, limited dissemination of information, and a concentration of services in large, urban hospitals. Individuals in higher social positions disproportionately benefit from their educational and economic advantages, which translate into higher levels of health literacy, greater capacity to acquire and interpret emerging health information, and stronger abilities to engage in proactive health management (Link and Phelan, 1995; Ross and Wu, 1995). In addition, their superior financial resources reduce barriers related to the affordability of newer and more expensive medical technologies. Through these mechanisms, socially advantaged groups are more likely to become early adopters of medical innovations, and health inequalities start to emerge and increase.

Then, as diffusion continues and reaches the early majority stage, medical innovations gradually spread beyond a small part of socioeconomically advantaged groups to broader segments of the population. While most high-SES individuals have already adopted these innovations, most of the lower-SES individuals still face a series of barriers related to economic cost, health information, and access, causing health inequalities to peak at the early majority stage.

Finally, as medical innovations become more widely available at the late majority and laggards stages, lower-SES groups gradually adopt them, leading to a substantial decline in health inequalities when diffusion reaches these later stages (Zapata-Moya et al., 2019, 2023; Weiss et al., 2020). This decline may result from reduced financial barriers due to decreasing costs over time, expanded health insurance coverage, and the integration of these innovations into basic public health services. Additionally, broader dissemination of health information through information diffusion contributes to increased awareness and uptake among disadvantaged groups.

However, convergence in inequality for a specific medical innovation does not translate to eliminating health inequalities at the population level. As new innovations emerge, high-SES groups benefiting from their flexible resources consistently sustain their advantage by adopting these health technologies earlier. In contrast, low-SES groups often face constraints that limit their ability to access such innovations at the same time as their high-SES counterparts. Following this logic, even when low-SES groups gradually benefit from the health innovations, and the older mechanisms generating health inequalities diminish or lose significance, the high-SES groups can always maintain their privileged position through the substitution of mechanisms. Therefore, such an evolving pattern of medical diffusion and related inequality dynamics together offer a valuable opportunity to test the meta-hypothesis of FCT.

Over the past few decades, China has made significant progress in expanding the coverage and availability of medical resources. The healthcare system has evolved from offering limited basic services, such as blood pressure testing, to implementing these services at scale and gradually introducing more specialized screenings, including cancer detection programs (Wang et al., 2019). Following the 2009 healthcare reform, the strengthening of the primary healthcare system and public health services substantially improved access to basic check-ups. Services such as blood pressure monitoring have become widely available through community-level health facilities, extending coverage to rural and low-income populations (Jakovljevic et al., 2017; Li and Fu, 2017). In contrast, more specialized screening programs, such as cancer screenings, while increasingly promoted alongside medical innovations, remain far less prevalent in terms of population-level diffusion compared to basic check-up programs (Xia et al., 2023), reflecting the fact that they are still at earlier stages of the diffusion cycle. This slower uptake is largely due to higher resource requirements and access barriers, including the need for advanced diagnostic technologies, specialized personnel, comprehensive follow-up care, and greater financial costs.

Given the increasing availability of health check-ups, which is influenced by both structural factors and flexible resource differentiation among urban and rural populations, and the dynamic nature of related inequalities, it is essential to explore how the expansion of medical resources interacts with the *hukou* system to perpetuate inequalities in health check-up utilization. Against this backdrop, and drawing on the framework of DOI, we argue that basic health services, including blood pressure tests, have progressed from the early stages of the diffusion cycle, such as innovators and early adopters, to more advanced stages like the late majority. Correspondingly, the associated health inequalities have followed a trajectory of emergence, expansion, and eventual decline. In contrast, some emerging specialized screening programs are still in the early stages of diffusion, during which related health inequalities tend to widen.

Although innovations in health check-ups have spread over time and urban–rural disparities in adoption of basic health check-ups may evolve through processes of emergence, expansion, decline, and eventual disappearance, due to the broad diffusion, urban *hukou* holders continue to sustain health advantages by accessing higher-quality health resources at earlier stages, owing to their privileged status, as we described in the previous section. Accordingly, we propose the following hypotheses:

*Hypothesis 3: In the early stages of health resource diffusion (such as innovators and early adopters stages), urban hukou holders are more likely to initiate health check-ups earlier than rural hukou holders, and such inequalities widen as diffusion progresses until reaching the early majority stages.*

*Hypothesis 4: As health resources become more widely available and less scarce (such as late majority and laggards stages), urban–rural inequalities in the initiation of health check-up services become less pronounced.*

## 3 Methods

### 3.1 Data

This study draws on the China Health and Retirement Longitudinal Study (CHARLS), a nationally representative survey of Chinese adults aged 45 and above, conducted by Peking University across 450 communities in 28 provinces (Zhao et al., 2014). We used data from the 2014 Life History survey, which provides retrospective life course information on health check-up history. The analytic sample was restricted to respondents with complete information on all predictors and then merged with each of the six health check-up outcomes, respectively. The final sample sizes were as follows: blood pressure test (*N* = 16,374), blood test (for glucose or cholesterol) (*N* = 17,214), dental exam (*N* = 18,032), vision exam (*N* = 17,724), breast screening (*N* = 9,307), and prostate cancer screening (*N* = 8,898). See Appendix Figure A1 for the detailed sample selection procedure.

### 3.2 Measures

#### 3.2.1 Dependent variables

This study analyzed six time-to-event outcomes, each capturing the duration from age 16 to the first uptake of a specific health check-up: blood pressure test, blood test (for glucose or cholesterol), vision exam, dental exam, breast screening, and prostate cancer screening. These outcomes were constructed based on responses to two survey questions: (1) “Did you have [the specific exam] after age 16?” and, if yes, (2) “In which year did you first have [the specific exam] after age 16?” Respondents who answered “yes” were coded as having used the specific health check-up (event = 1), whereas those who answered “no” were treated as right censored at the survey year (event = 0), indicating that the first uptake had not occurred by the time of the survey. Survival time was defined as the number of years from age 16 to the year of first uptake of the specific health check-up for those with an observed event, and to the survey year for censored respondents.

#### 3.2.2 Urban–rural classification

This key predictor was constructed by combining *hukou* status and place of residence, as summarized in Table 1, and categorized into three groups: rural residents with rural *hukou*, urban residents with rural *hukou*, and urban *hukou* holders. Respondents with a lifelong rural *hukou* were classified as rural *hukou* holders, whereas those with a lifelong urban *hukou* or who had converted from rural to urban *hukou* were classified as urban *hukou* holders. Based on place of residence at the time of the survey, respondents living in rural villages were coded as rural residents, while those residing in urban communities were coded as urban residents. Given the small number of urban *hukou* holders residing in rural areas and the study’s focus on *hukou* as an institutional determinant of health screening uptake, urban *hukou* holders were not further disaggregated by place of residence.

**Table 1:** Urban–rural classification.

Place of residence	*Hukou* status	
	Urban *hukou* holders	Rural *hukou* holders
Living in the urban community	Urban *hukou* holders	Urban residents with rural *hukou*
Living in the rural village	Urban *hukou* holders	Rural residents with rural *hukou*

#### 3.2.3 Covariates

We adjusted for a range of potential confounders that may influence both urban–rural classification and health exam outcomes (Qin and Hsieh, 2014; Gong et al., 2016). Demographic variables included gender (male = 0, female = 1), birth cohort (1917–1948, 1949–1958, 1959–1969), and marital status which was measured as a binary variable. Respondents were classified as continuously married if they remained married to their first spouse, otherwise coded as non-continuously married, including those who were never married or had ever experienced marital dissolution, regardless of remarriage. For socioeconomic status, we adjusted for educational attainment (illiterate, less than middle school, middle school, high school, and college or above), and primary occupation across the life course, defined as the work type in which the respondent spent the most years and categorized as economic inactivity, agricultural work, or non-agricultural work. We also included hospitalization experience during adulthood as a binary variable, indicating whether the respondent had ever been hospitalized or not. Finally, regional residence was controlled using a three-category variable (eastern coastal, central, or western inland economic region) to account for disparities in regional development.

### 3.3 Statistical analysis

We first estimated Kaplan–Meier (KM) curves for each of the six health check-up outcomes. For each outcome, KM curves were stratified by urban–rural classification, and Wilcoxon tests were used to assess the statistical significance of survival differences across urban–rural groups. In this context, the survival probability represents the proportion of respondents who had not yet received the health check-up at a given time point, such that higher survival probabilities indicate longer delays. Given violations of the proportional hazards assumption in the Cox model, we adopted accelerated failure time (AFT) models to adjust for potential confounders and examine urban–rural differences in time to first uptake of the specific health check-up. Model estimates were reported as time ratios (TR) with corresponding 95% confidence intervals (CI). AFT models were estimated using four distributions, including Weibull, log-logistic, log-normal, and exponential, with the best-fitting model selected based on the lowest Akaike information criterion (AIC) value. All models were estimated using the *survival* package in R. To assess potential heterogeneity, we further fitted separate models by birth cohort and geographic region.

To account for unobserved heterogeneity across provinces, such as variation in urbanization, healthcare infrastructure, and service quality, we incorporated province fixed effects as a robustness strategy to address potential confounding arising from contextual differences. Furthermore, among older age groups, differential mortality related to urban–rural classification and health screening uptake may introduce sample selection bias by disproportionately excluding rural residents and individuals with lower and delayed uptake. To assess the robustness of our findings to this potential bias, we conducted a robustness check restricted to individuals aged 45 to 74, which falls below China’s 2013 average life expectancy of 75 years (World Health Organization, 2015).

## 4 Results

**Figure 1:**
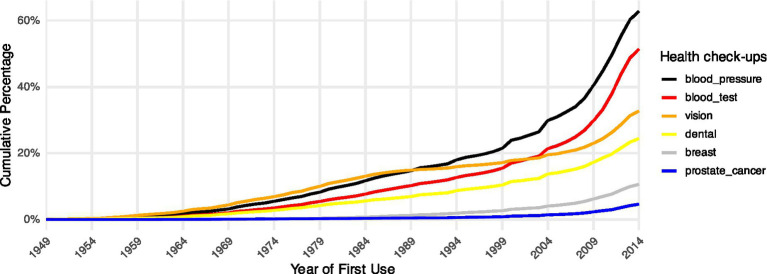
Cumulative percentage of first uptake for each type of health check-up, by year.

Figure 1 presents the cumulative percentage of individuals who underwent their first health check-up between 1949 and 2014. Over this period, all types of health check-ups experience an increase in uptake, reflecting a general trend of progressive diffusion at the population level. Notably, basic screening services, such as blood pressure and blood tests, consistently exhibit the highest cumulative uptake rates, with a sharp acceleration since 2004. This surge aligns with the expansion of primary healthcare services, suggesting a significant reduction in access barriers. This pattern indicates that these services have reached the advanced stage of the diffusion cycle at the population level. In contrast, utilization levels of screenings with higher access thresholds, such as breast and prostate cancer screenings, remain substantially lower, showing that these services are still in the early stages of the diffusion cycle at the population level.

**Figure 2:**
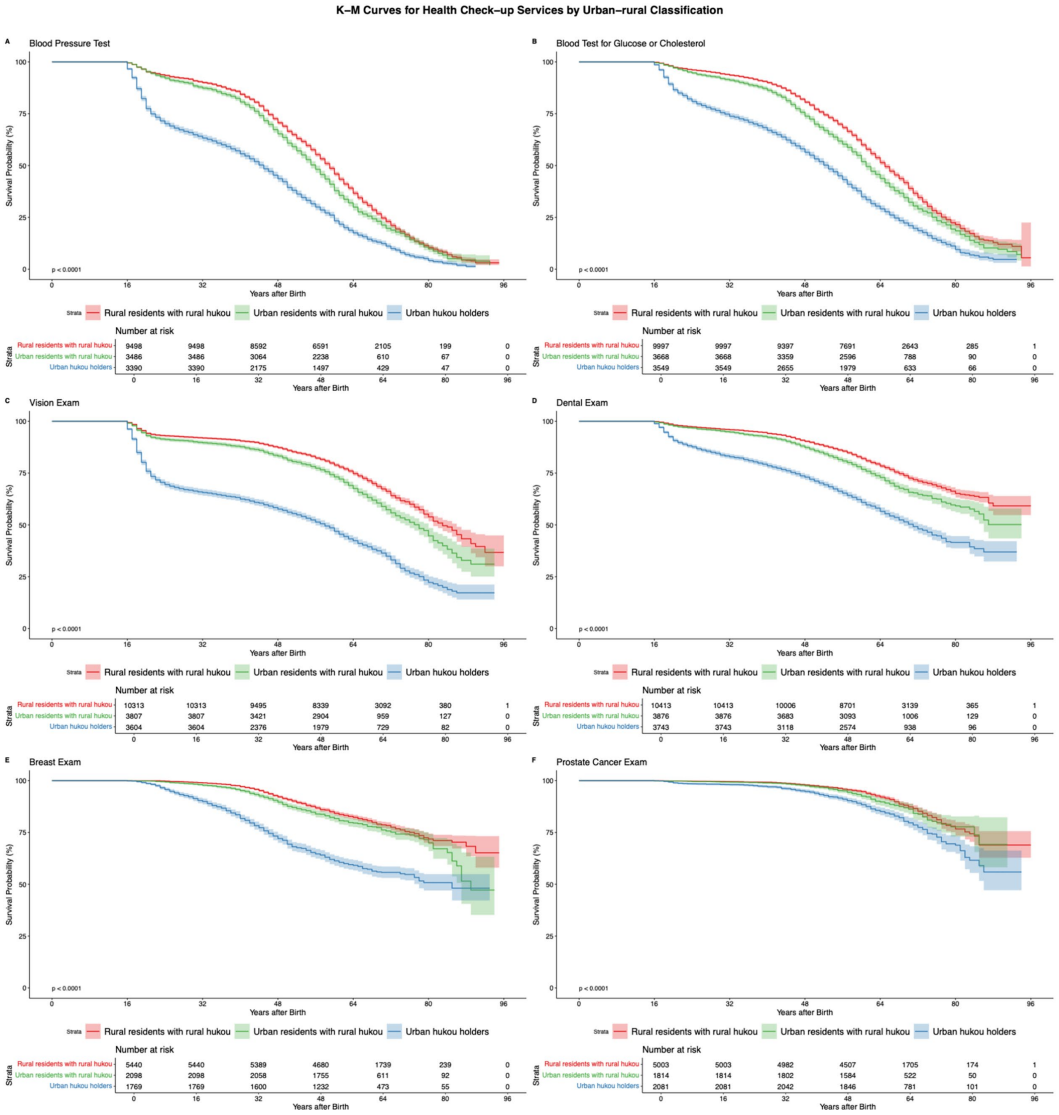
**(A-F)** KM curves for duration before the first health screening (by age).

Appendix Table A1 displays bivariate analyses by urban–rural classification across different types of health check-ups. Urban *hukou* holders consistently demonstrate higher uptake rates and earlier median timing of first use across all outcomes. Among rural *hukou* holders, those residing in urban areas exhibit modest advantages in certain screenings, particularly blood pressure and blood tests. Figure 2 presents Kaplan–Meier curves for time to first health check-up, stratified by urban–rural classification. Higher survival probabilities indicate a lower cumulative proportion of individuals who have undergone their first check-up by each age. The curves reveal significant disparities between urban and rural groups. Urban *hukou* holders consistently initiate screenings earlier than their rural counterparts, although the magnitude and pattern of disparities vary by screening type. For basic services that have reached the advanced stage of the diffusion cycle, such as blood pressure and blood tests, urban–rural inequalities tend to narrow in later life. In contrast, for services that remain in the early stages of diffusion, such as breast and prostate cancer screenings, inequalities become more pronounced during middle and later adulthood. Appendix Figures A2–A7 display K-M curves based on subsamples stratified by birth cohort and geographic region, further confirming the consistency of the observed patterns.

Table 2 presents the results from the accelerated failure time models to further adjust for potential confounders and examine urban–rural inequalities in the duration in the timing of first health check-up use. Compared to urban residents with rural *hukou*, urban *hukou* holders experience consistently shorter durations before initiating health screenings. The most substantial disparity is observed for vision exam, where the duration without screening is 21.7% shorter (TR = 0.783), followed by dental exam (15.2%, TR = 0.848), breast screening (10.8%, TR = 0.892), blood pressure test (10.7%, TR = 0.893), blood test (8.1%, TR = 0.919), and prostate cancer screening (6.7%, TR = 0.933). Results using rural residents with rural *hukou* as the reference group are presented in Appendix Table A2, confirming the robustness of the above findings. These results demonstrate a consistent pattern of earlier initiation of health check-up services among urban *hukou* populations, which aligns with *Hypothesis 1*.

**Table 2:** AFT models for duration before the first health check-ups.

Predictors	Blood pressure test			Blood test			Breast screening			Prostate cancer screening			Dental exam			Vision exam		
	TR	95% CI	Sig.	TR	95% CI	Sig.	TR	95% CI	Sig.	TR	95% CI	Sig.	TR	95% CI	Sig.	TR	95% CI	Sig.
Urban–rural (Ref = Urban residents with rural *hukou*)																		
Rural residents with rural *hukou*	1.012	[0.999, 1.025]		1.028	[1.014, 1.042]	***	0.998	[0.972, 1.025]		1.015	[0.979, 1.052]		1.062	[1.032, 1.094]	***	1.083	[1.048, 1.120]	***
Urban *hukou* holders	0.893	[0.879, 0.908]	***	0.919	[0.904, 0.934]	***	0.892	[0.865, 0.920]	***	0.933	[0.896, 0.971]	***	0.848	[0.820, 0.877]	***	0.783	[0.754, 0.812]	***
Education (Ref = Illiterate)																		
Less than Middle school	0.934	[0.921, 0.947]	***	0.935	[0.921, 0.948]	***	0.940	[0.914, 0.968]	***	0.954	[0.917, 0.992]	*	0.890	[0.862, 0.919]	***	0.838	[0.807, 0.869]	***
Middle school	0.888	[0.874, 0.902]	***	0.896	[0.881, 0.910]	***	0.865	[0.839, 0.891]	***	0.961	[0.920, 1.004]		0.851	[0.822, 0.881]	***	0.728	[0.699, 0.757]	***
High school	0.834	[0.818, 0.850]	***	0.858	[0.841, 0.875]	***	0.800	[0.772, 0.830]	***	0.950	[0.904, 0.998]	*	0.767	[0.736, 0.798]	***	0.582	[0.556, 0.609]	***
College and above	0.702	[0.680, 0.724]	***	0.721	[0.699, 0.743]	***	0.639	[0.607, 0.673]	***	0.843	[0.793, 0.895]	***	0.636	[0.601, 0.674]	***	0.423	[0.397, 0.451]	***
Marital (Ref = Continuously married)																		
Ever disrupted or never married	1.030	[1.016, 1.044]	***	1.041	[1.027, 1.056]	***	1.073	[1.043, 1.104]	***	1.046	[1.009, 1.085]	*	1.037	[1.007, 1.068]	*	0.992	[0.960, 1.025]	
Gender (ref = Male)																		
Female	0.995	[0.984, 1.006]		0.979	[0.968, 0.990]	***	/	/	/	/	/	/	0.946	[0.924, 0.968]	***	1.171	[1.140, 1.202]	***
Main work (Ref = Non-agricultural)																		
Economic inactivity	1.058	[1.038, 1.080]	***	1.068	[1.046, 1.090]	***	1.113	[1.075, 1.151]	***	1.055	[0.993, 1.121]		1.096	[1.051, 1.143]	***	1.172	[1.118, 1.228]	***
Agricultural labor	1.037	[1.023, 1.051]	***	1.049	[1.035, 1.064]	***	1.099	[1.069, 1.129]	***	1.007	[0.977, 1.038]		1.064	[1.035, 1.094]	***	1.141	[1.107, 1.176]	***
Hospitalization in adulthood (Ref = No)																		
At least once	0.930	[0.917, 0.944]	***	0.895	[0.882, 0.907]	***	0.943	[0.915, 0.972]	***	0.933	[0.905, 0.962]	***	0.912	[0.885, 0.940]	***	0.880	[0.851, 0.909]	***
Birth Group (Ref = 1917–1948)																		
Birth cohort: 1949–1958	0.850	[0.839, 0.861]	***	0.856	[0.844, 0.867]	***	0.782	[0.759, 0.807]	***	0.900	[0.872, 0.928]	***	0.859	[0.836, 0.884]	***	0.906	[0.878, 0.934]	***
Birth cohort: 1959–1969	0.728	[0.718, 0.739]	***	0.736	[0.725, 0.747]	***	0.613	[0.594, 0.632]	***	0.794	[0.765, 0.823]	***	0.787	[0.763, 0.811]	***	0.850	[0.822, 0.880]	***
Region (Ref = East)																		
Middle	1.029	[1.016, 1.042]	***	1.016	[1.003, 1.028]	*	1.090	[1.064, 1.116]	***	1.004	[0.973, 1.037]		1.102	[1.073, 1.131]	***	1.110	[1.078, 1.144]	***
West	1.038	[1.025, 1.051]	***	1.017	[1.005, 1.031]	**	1.061	[1.036, 1.087]	***	0.985	[0.955, 1.016]		1.093	[1.064, 1.123]	***	1.051	[1.021, 1.083]	***
Intercept	73.003	[71.441, 74.598]	***	79.896	[78.136, 81.695]	***	106.447	[101.727, 111.387]	***	112.087	[105.570, 119.006]	***	116.568	[111.100, 122.305]	***	106.654	[101.171, 112.434]	***
Log(scale)	0.275	[0.271, 0.280]	***	0.256	[0.252, 0.261]	***	0.221	[0.212, 0.230]	***	0.189	[0.177, 0.201]	***	0.369	[0.359, 0.380]	***	0.469	[0.458, 0.480]	***
AIC	104898.7			91149.2			21756.4			11073.0			53008.3			64937.3		
Sample size	16,374			17,214			9,307			8,898			18,032			17,724		

*p < 0.05; **p < 0.01; ***p < 0.001. The results are derived from Accelerated Failure Time (AFT) Weibull models. TR is the Time Ratio from AFT models. The reported coefficients for Log(scale) are exp(log(scale)), representing the scales of AFT models.

In addition, among rural *hukou* holders, rural residents experience significantly longer durations before initiating several types of health screenings than their urban counterparts, specifically for blood test (TR = 1.028), dental exam (TR = 1.062), and vision exam (TR = 1.083). These results indicate delays in screening uptake among rural residents, providing partial support for *Hypothesis 2* and suggesting an urban spillover effect, where proximity to urban areas facilitates earlier uptake.

Moreover, the findings show that inequalities associated with *hukou* consistently and substantially exceed those linked to geographic residence. Even for screenings with relatively high diffusion, such as blood test, urban *hukou* holders initiate screening nearly 10% earlier than urban residents with rural *hukou* (TR = 0.919). In contrast, the latter group initiates screening only about 3% earlier than rural residents with rural *hukou* (TR = 1.028). These patterns underscore that *hukou* as an institutional arrangement serves as more fundamental determinant of health service utilization than geographic factors alone.

Based on Figure 2, for less diffused health check-ups like breast and prostate cancer screenings, urban *hukou* holders tend to initiate use earlier, and *hukou*-based inequalities intensify as these services spread. This pattern implies that as emerging health innovations gradually spread, access remains selective, thereby reproducing new inequalities in health check-ups. Further comparison of the coefficients for *hukou*-based inequalities in cancer screenings with those for dental and vision examinations indicates that health check-ups at an early stage of the diffusion cycle exhibit smaller inequalities, which further supports *Hypothesis 3*. In contrast, for health check-ups that have reached an advanced stage of diffusion, such as blood pressure tests and blood tests for glucose or cholesterol, we find a convergence in urban–rural inequalities in first-time uptake during the later stages of the life course. This suggests that as these resources become more widely available, inequalities in initiation diminish. Further comparison of the coefficients for *hukou*-based inequalities in blood pressure and blood tests with those for dental and vision examinations indicates that health check-ups at a more advanced stage of the diffusion cycle exhibit smaller inequalities, which further supports *Hypothesis* 4. Notably, the comparable magnitude of *hukou*-based inequalities between highly diffused services such as blood pressure and blood tests and less diffused cancer screenings, alongside the larger inequalities observed for intermediately diffused services such as dental and vision exams, together reproduce the full inverted U-shaped inequality pattern predicted by the hypothetical pattern proposed by Zapata-Moya et al. (2019), providing empirical evidence on how mechanism substitution unfolds.

Appendix Figure A8 presents findings on regional and cohort heterogeneity in urban–rural inequalities in the duration until first health screening. Detailed results are provided in Appendix Figures A2–A7 (KM curves) and Appendix Tables A5–A10 (AFT models). Robustness checks stratified by sex for blood pressure tests, cholesterol tests, dental exams, and vision exams were also conducted, with results consistent across male and female subsamples (see Appendix Tables A13, A14). For the 1917–1948 cohort, no significant *hukou* inequalities are observed in early diffusion services, such as breast and prostate cancer screenings. In contrast, *hukou* inequalities in these services are more pronounced in the eastern region. Aside from these specific variations, the overall patterns remain broadly consistent with the main analysis.

To assess sensitivity to distributional assumptions, we additionally estimated AFT models using alternative parametric distributions and compared model fit using AIC (Appendix Table A3). Only three outcomes were better fitted by non-Weibull specifications, and the corresponding estimates are reported in Appendix Table A4. The substantive findings remain unchanged across distributions. Furthermore, robustness checks addressing potential mortality selection and provincial confounding are presented in Appendix Tables A11, A12, respectively. These additional analyses indicate that neither mortality selection nor provincial-level confounding materially alters our conclusions. Overall, the main findings persist across most robustness checks.

## 5 Conclusion and discussion

This study reveals persistent health inequities in the timing of initial health check-ups across urban–rural populations. Although urban residence, to some extent, offers health spillover benefits, such as greater proximity to health services, increased awareness, and economic affordability, facilitating earlier uptake of some health check-ups among rural *hukou* holders residing in urban areas than those living in rural areas. Our findings suggest that even rural *hukou* holders who have settled in urban areas continue to experience delays in accessing health check-up services compared to their urban *hukou* counterparts. This pattern indicates that the *hukou* identity remains a salient institutional barrier for rural *hukou* holders to accessing health services, which is in line with prior research (Li et al., 2018; Song and Smith, 2019). This finding underscores the *hukou* system as a structural determinant of health that exerts a more profound influence than place of residence in shaping health inequalities (Dorélien and Xu, 2020; Chen et al., 2025). In particular, entrenched social stratification and institutional discrimination, such as occupation segregation, differentiated access to medical insurance and pension schemes, are embedded within the *hukou* system and systematically marginalize rural *hukou* holders within healthcare systems and play a central role in producing and perpetuating urban–rural health inequalities (Afridi et al., 2015; Wu, 2019; Song and Smith, 2021).

For screenings with higher population-level diffusion, such as blood pressure and blood tests, urban–rural inequalities have narrowed as these services have become more widely available. The widespread adoption of these basic screenings may be attributed to several enabling factors, including high public awareness of associated conditions, particularly cardiovascular disease risk factors, relatively low diagnosis complexity, and lower financial cost. These features may increase willingness to participate and reduce access barriers, thereby contributing to the narrowing of urban–rural disparities among older adults. In addition, universal public health programs, such as the National Essential Public Health Services, may also promote equitable access to basic preventive care (Zhang et al., 2018), particularly for financially constrained older rural *hukou* holders (Xu et al., 2022). By contrast, for health check-ups with limited diffusion, such as breast and prostate cancer screenings, *hukou*-related inequalities tend to widen as these services spread. In the absence of universal policy support, access to these services relies more heavily on individuals’ flexible resources. Urban *hukou* holders benefit from cumulative advantages, including superior insurance coverage, greater economic resources, and better health literacy. These factors are likely to enhance both their willingness and capacity to initiate these health services at earlier stages. In contrast, rural *hukou* holders are more likely to delay or forgo use due to limited health literacy and constrained access (Long et al., 2022; Yu et al., 2022).

The inequality patterns we observed align with prior work based in Spain by Zapata-Moya et al. (2019), which documents larger socioeconomic inequalities for preventive practices, such as prostate-specific antigen tests and mammograms, at earlier diffusion stages, including early and late majority, and smaller inequalities once uptake becomes widespread in the late majority or laggards stages, as observed for services like blood test for glucose or cholesterol and blood-pressure checks. This consistency across contexts supports the generalizability of the diffusion of innovations framework for investigating how socioeconomic inequalities vary across stages of diffusion, illustrating how new preventive services can serve as replacement mechanisms that sustain health inequalities over time.

Drawing on the fundamental cause perspective, the *hukou* system does not reproduce health inequalities through any single fixed mechanism, but rather by continuously mobilizing sets of flexible resources across successive medical innovations, so that new mechanisms emerge to replace older ones that produce health inequalities. As demonstrated in this study, when the six services are ordered by population-level diffusion, the magnitude of hukou-based inequalities follows an inverted U-shaped pattern, with comparable inequalities observed at both ends of the diffusion spectrum, highly diffused services such as blood pressure and blood tests on one end and less diffused cancer screenings on the other, and the largest inequalities found for intermediately diffused services such as vision and dental exams. This pattern mirrors the full inequality trajectory predicted by the hypothetical pattern proposed by Zapata-Moya et al. (2019) and provides coherent empirical support for the operation of mechanism substitution. Overall, as long as substitution mechanisms continue to operate, the emergence of new health screenings alongside the widespread diffusion of older services is likely to reproduce *hukou*-related health inequalities. Advantaged urban *hukou* holders are positioned to access and initiate newly introduced screenings at earlier stages of diffusion, benefiting from higher health literacy, greater economic resources, more favorable insurance entitlements, and closer geographic access to high-quality healthcare. In contrast, disadvantaged rural *hukou* holders experience delayed uptake, leading to the continual reproduction of *hukou*-based inequalities in health check-up access.

The absence of *hukou* inequalities in prostate cancer and breast exams among the 1917–1948 cohort may reflect cohort-specific timing and selection processes. When breast and prostate cancer screenings began to diffuse, individuals in this cohort had already reached older ages, and overall participation remained relatively low, potentially limiting the extent to which inequalities could emerge. In addition, survivorship to the survey period may positively select individuals with relatively better health and socioeconomic resources, thereby attenuating observed urban–rural inequalities. In addition, the more pronounced *hukou* inequalities observed in the eastern region may reflect greater resource availability and a more advanced stage of service diffusion, particularly for screenings that lack organized or universal provision, such as breast and prostate cancer exams. For these services, uptake depends more heavily on individual-level flexible resources, potentially leading to earlier adoption among urban *hukou* holders.

To reduce inequalities in health check-up uptake between urban and rural populations, addressing the persistent institutional inequalities caused by the *hukou* system should be a core policy objective. This requires reforms aimed at promoting urban–rural healthcare integration, expanding social welfare coverage, and establishing an equitable pension scheme to alleviate financial burdens on rural *hukou* holders. Policymakers should also prioritize tailored health campaigns to raise awareness, while updating healthcare infrastructure and increasing the number of skilled professionals in rural areas. These efforts should be further differentiated according to the diffusion stage of each health check-up. For high-diffusion services such as blood pressure and blood tests, where urban–rural inequalities have begun to narrow, policy efforts should continue to prioritize reducing remaining gaps, with targeted support for rural *hukou* holders to improve their access and uptake further. For low-diffusion services such as breast and prostate cancer exams, where urban–rural gaps are widening, rural *hukou* holders should be placed at the center of policy responses to prevent further deterioration of health inequalities. This requires financial subsidies, organized screening programs, and dedicated outreach campaigns, with particular attention to rural populations in central and western regions.

While the overall pattern of our findings aligns with the theoretical predictions of the FCT-DOI framework, several critical reflections are warranted. First, the classification of health check-up services into specific diffusion stages rests on theoretical inference rather than clear and validated criteria, as the framework specifies no precise number above which a diffusion of a specific health service formally enters a given stage in the DOI. Such classifications are therefore inevitably approximate, and future research would benefit from developing externally validated indicators to place stage assignment on more systematic grounds. Second, the framework assumes relatively uniform predictions about inequality trajectories across diffusion stages. However, the pace and magnitude of inequality change may vary considerably depending on institutional context (De Prez et al., 2021). Institutional interventions may compress the inequality-widening phase for services with organized provision, while their absence may prolong and amplify inequalities for the emerging services. The FCT-DOI framework has yet to fully theorize and develop how institutional arrangements condition the substitution process itself. Third, the assumption that inequalities converge as diffusion continues may not apply equally across all health service types. For screenings for several chronic non-communicable diseases, individual flexible resources continue to be highly linked to access even at extremely high diffusion levels, suggesting that inequalities may persist. For instance, some people with low SES still face a high risk of cardiovascular diseases due to delayed or inadequate screening, even if the diffusion of these exams is high. However, for infectious disease vaccines, herd immunity may fundamentally alter the inequality dynamic, as unvaccinated individuals can still benefit from reduced population-level risk and the extension of the protection (Fine, 1993). Therefore, the applicability of the framework may need to be considered in light of the nature of the health service in question.

Besides the above-mentioned theoretical limitations, this study is subject to several limitations in the empirical analysis. First, the outcomes rely on respondents’ retrospective self-reports, which are inherently susceptible to recall bias and may compromise measurement validity. While we conducted sensitivity analyses restricted to respondents under age 75, this subsample is not immune to recall bias, as accurately recalling the timing of health check-ups that occurred decades earlier remains challenging. Importantly, recall bias may differ across urban and rural populations. For instance, urban *hukou* holders may more easily remember early check-ups due to higher participation in employer-organized health screenings with institutional documentation. If such differential recall exists, the urban–rural inequalities observed in this study may in fact be underestimated. Future research could address this limitation by validating self-reported health service use against external administrative or medical records where feasible. Second, given urban–rural disparities in mortality, individuals in poorer health or with limited access to health check-ups, particularly rural residents, are less likely to survive until the survey year, leading to selection bias and an underestimation of true health inequalities (Zimmer et al., 2007). Although we conducted sensitivity analyses to assess the robustness of our findings, this inherent limitation of retrospective data may still bias our estimates. To better address this issue, future studies could apply inverse probability weighting or employ prospective cohort designs. Third, while this study identifies urban–rural disparities in health service uptake, it does not empirically examine the underlying mechanisms. Investigating potential mediators such as health insurance coverage and healthcare accessibility would contribute to a more comprehensive understanding. Additionally, this study does not fully capture life course patterns of marriage, work, *hukou*, and residential histories, which recent research shows influence on health outcomes among older Chinese adults (Zhang et al., 2021; Wang et al., 2025). These patterns are also linked to urban–rural classification and health check-up use and therefore may confound the observed associations. Future research could apply sequence analysis to better capture these dynamic influences. Finally, it should be noted that the present study uses six health check-up services at different stages of diffusion as a proxy to approximate the trajectory of health inequalities, rather than directly tracking changes within any given service across the full diffusion cycle. A rigorous test of mechanism substitution would still require a longitudinal research design.

## Data Availability

The data used in this study are publicly accessible, and the code for data cleaning and analysis is available from the corresponding author upon request.
