# Supplementary material for: The line is drawn, the fate is cast: urban–rural inequalities in the timing of initial health check-ups in China
Source: Front Sociol. 2026 May 8;11:1702900. doi: 10.3389/fsoc.2026.1702900 (PMC13193861; doi:10.3389/fsoc.2026.1702900)
Supplement: Supplementary file 1 [file Supplementary_file_1.DOCX]

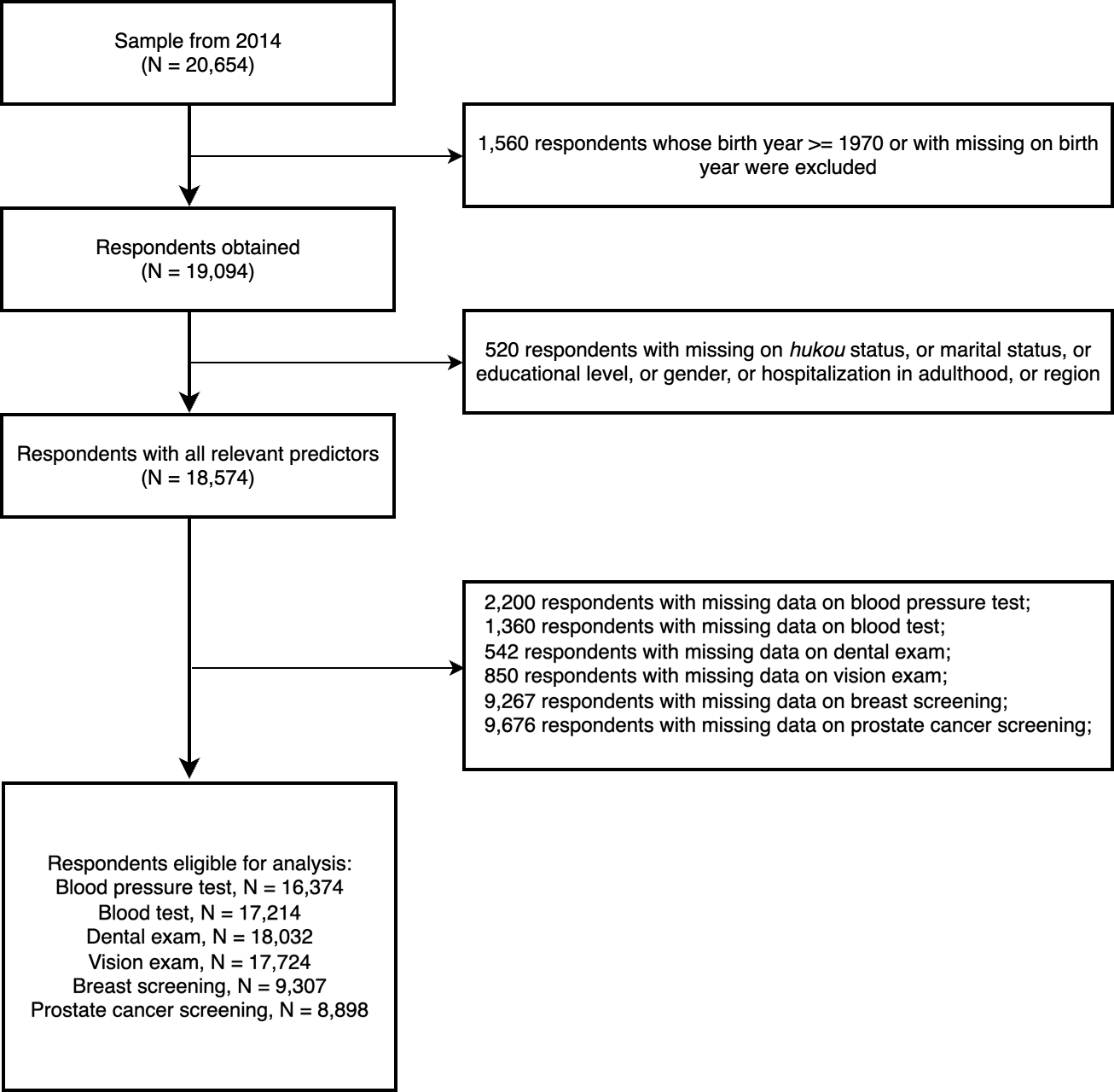


Figure A1. Sample selection procedure

Table A1. Sample Characteristics by Urban-rural Classification

| **Predictors** | **Blood Pressure Test** | | |  | **Blood Test** | | |  |
| --- | --- | --- | --- | --- | --- | --- | --- | --- |
|  | **Rural residents with rural *hukou*** | **Urban residents with rural *hukou*** | **Urban *hukou* holders** |  | **Rural residents with rural *hukou*** | **Urban residents with rural *hukou*** | **Urban *hukou* holders** |  |
|  | (N = 9,498) | (N = 3,486) | (N = 3,390) | *p-value* | (N = 9,997) | (N = 3,668) | (N = 3,549) | *p-value* |
| *Education* |  |  |  | < 0.001 |  |  |  | < 0.001 |
| Illiterate | 3,929 (41.4%) | 1,095 (31.4%) | 382 (11.3%) |  | 4,119 (41.2%) | 1,154 (31.5%) | 402 (11.3%) |  |
| Less than middle school | 2,823 (29.7%) | 1,119 (32.1%) | 734 (21.7%) |  | 2,962 (29.6%) | 1,160 (31.6%) | 763 (21.5%) |  |
| Middle school | 2,099 (22.1%) | 959 (27.5%) | 1,036 (30.6%) |  | 2,232 (22.3%) | 1,010 (27.5%) | 1,084 (30.5%) |  |
| High School | 621 (6.5%) | 284 (8.1%) | 876 (25.8%) |  | 658 (6.6%) | 309 (8.4%) | 922 (26.0%) |  |
| College and above | 26 (0.3%) | 29 (0.8%) | 362 (10.7%) |  | 26 (0.3%) | 35 (1.0%) | 378 (10.7%) |  |
| *Marital status* |  |  |  | 0.257 |  |  |  | 0.197 |
| Continuously married | 7,705 (81.1%) | 2,868 (82.3%) | 2,778 (81.9%) |  | 8,109 (81.1%) | 3,019 (82.3%) | 2,912 (82.1%) |  |
| Ever disrupted or never married | 1,793 (18.9%) | 618 (17.7%) | 612 (18.1%) |  | 1,888 (18.9%) | 649 (17.7%) | 637 (17.9%) |  |
| *Gender* |  |  |  | < 0.001 |  |  |  | < 0.001 |
| Male | 4,523 (47.6%) | 1,596 (45.8%) | 1,817 (53.6%) |  | 4,783 (47.8%) | 1,677 (45.7%) | 1,897 (53.5%) |  |
| Female | 4,975 (52.4%) | 1,890 (54.2%) | 1,573 (46.4%) |  | 5,214 (52.2%) | 1,991 (54.3%) | 1,652 (46.5%) |  |
| *Main work* |  |  |  | < 0.001 |  |  |  | < 0.001 |
| Non-agricultural labor | 1,080 (11.4%) | 846 (24.3%) | 1,856 (54.7%) |  | 1,152 (11.5%) | 896 (24.4%) | 1,932 (54.4%) |  |
| Economic inactivity | 849 (8.9%) | 371 (10.6%) | 385 (11.4%) |  | 907 (9.1%) | 394 (10.7%) | 420 (11.8%) |  |
| Agricultural labor | 7,569 (79.7%) | 2,269 (65.1%) | 1,149 (33.9%) |  | 7,938 (79.4%) | 2,378 (64.8%) | 1,197 (33.7%) |  |
| *Hospitalization in adulthood* |  |  |  | < 0.001 |  |  |  | < 0.001 |
| No | 8,348 (87.9%) | 3,088 (88.6%) | 2,840 (83.8%) |  | 8,791 (87.9%) | 3,250 (88.6%) | 2,977 (83.9%) |  |
| At least once | 1,150 (12.1%) | 398 (11.4%) | 550 (16.2%) |  | 1,206 (12.1%) | 418 (11.4%) | 572 (16.1%) |  |
| *Birth Group* |  |  |  | < 0.001 |  |  |  | < 0.001 |
| 1917-1948 | 2,594 (27.3%) | 827 (23.7%) | 1,082 (31.9%) |  | 2,751 (27.5%) | 863 (23.5%) | 1,121 (31.6%) |  |
| 1949-1958 | 3,431 (36.1%) | 1,235 (35.4%) | 1,200 (35.4%) |  | 3,578 (35.8%) | 1,300 (35.4%) | 1,254 (35.3%) |  |
| 1959-1969 | 3,473 (36.6%) | 1,424 (40.8%) | 1,108 (32.7%) |  | 3,668 (36.7%) | 1,505 (41.0%) | 1,174 (33.1%) |  |
| *Region* |  |  |  | < 0.001 |  |  |  |  |
| East | 3,094 (32.6%) | 1,390 (39.9%) | 1,026 (30.3%) |  | 3,304 (33.0%) | 1,445 (39.4%) | 1,091 (30.7%) |  |
| Middle | 2,908 (30.6%) | 916 (26.3%) | 1,386 (40.9%) |  | 3,088 (30.9%) | 994 (27.1%) | 1,442 (40.6%) |  |
| West | 3,496 (36.8%) | 1,180 (33.8%) | 978 (28.8%) |  | 3,605 (36.1%) | 1,229 (33.5%) | 1,016 (28.6%) |  |
| *Check-up use (binary)* |  |  |  | < 0.001 |  |  |  | < 0.001 |
| No | 3,276 (34.5%) | 1,077 (30.9%) | 503 (14.8%) |  | 5,112 (51.1%) | 1,662 (45.3%) | 971 (27.4%) |  |
| Yes | 6,222 (65.5%) | 2,409 (69.1%) | 2,887 (85.2%) |  | 4,885 (48.9%) | 2,006 (54.7%) | 2,578 (72.6%) |  |
| *Initial use (median)* | 2009 | 2007 | 1994 | < 0.001 | 2010 | 2009 | 1999 | < 0.001 |
| **Predictors** | **Dental Exam** | | |  | **Vision Exam** | | |  |
|  | **Rural residents with rural *hukou*** | **Urban residents with rural *hukou*** | **Urban *hukou* holders** |  | **Rural residents with rural *hukou*** | **Urban residents with rural *hukou*** | **Urban *hukou* holders** |  |
|  | (N = 10,413) | (N = 3,876) | (N = 3,743) | *p-value* | (N = 10,313) | (N = 3,807) | (N = 3,604) | *p-value* |
| *Education* |  |  |  | < 0.001 |  |  |  | < 0.001 |
| Illiterate | 4,290 (41.2%) | 1,226 (31.6%) | 431 (11.5%) |  | 4,292 (41.6%) | 1,224 (32.2%) | 417 (11.6%) |  |
| Less than middle school | 3,070 (29.5%) | 1,219 (31.4%) | 800 (21.4%) |  | 3,040 (29.5%) | 1,216 (31.9%) | 797 (22.1%) |  |
| Middle school | 2,335 (22.4%) | 1,067 (27.5%) | 1,143 (30.5%) |  | 2,287 (22.2%) | 1,030 (27.1%) | 1,098 (30.5%) |  |
| High School | 692 (6.6%) | 330 (8.5%) | 973 (26.0%) |  | 667 (6.5%) | 307 (8.1%) | 923 (25.6%) |  |
| College and above | 26 (0.2%) | 34 (0.9%) | 396 (10.6%) |  | 27 (0.3%) | 30 (0.8%) | 369 (10.2%) |  |
| *Marital status* |  |  |  | 0.114 |  |  |  | 0.040 |
| Continuously married | 8,409 (80.8%) | 3,178 (82.0%) | 3,068 (82.0%) |  | 8,311 (80.6%) | 3,119 (81.9%) | 2,964 (82.2%) |  |
| Ever disrupted or never married | 2,004 (19.2%) | 698 (18.0%) | 675 (18.0%) |  | 2,002 (19.4%) | 688 (18.1%) | 640 (17.8%) |  |
| *Gender* |  |  |  | < 0.001 |  |  |  | < 0.001 |
| Male | 4,969 (47.7%) | 1,792 (46.2%) | 2,008 (53.6%) |  | 4,886 (47.4%) | 1,739 (45.7%) | 1,935 (53.7%) |  |
| Female | 5,444 (52.3%) | 2,084 (53.8%) | 1,735 (46.4%) |  | 5,427 (52.6%) | 2,068 (54.3%) | 1,669 (46.3%) |  |
| *Main work* |  |  |  | < 0.001 |  |  |  | < 0.001 |
| Non-agricultural labor | 1,216 (11.7%) | 946 (24.4%) | 2,049 (54.7%) |  | 1,191 (11.5%) | 910 (23.9%) | 1,953 (54.2%) |  |
| Economic inactivity | 978 (9.4%) | 419 (10.8%) | 437 (11.7%) |  | 966 (9.4%) | 412 (10.8%) | 422 (11.7%) |  |
| Agricultural labor | 8,219 (78.9%) | 2,511 (64.8%) | 1,257 (33.6%) |  | 8,156 (79.1%) | 2,485 (65.3%) | 1,229 (34.1%) |  |
| *Hospitalization in adulthood* |  |  |  | < 0.001 |  |  |  |  |
| No | 9,143 (87.8%) | 3,433 (88.6%) | 3,120 (83.4%) |  | 9,055 (87.8%) | 3,366 (88.4%) | 3,008 (83.5%) |  |
| At least once | 1,270 (12.2%) | 443 (11.4%) | 623 (16.6%) |  | 1,258 (12.2%) | 441 (11.6%) | 596 (16.5%) |  |
| *Birth Group* |  |  |  | < 0.001 |  |  |  | < 0.001 |
| 1917-1948 | 2,897 (27.8%) | 944 (24.4%) | 1,176 (31.4%) |  | 2,876 (27.9%) | 935 (24.6%) | 1,155 (32.0%) |  |
| 1949-1958 | 3,688 (35.4%) | 1,349 (34.8%) | 1,325 (35.4%) |  | 3,674 (35.6%) | 1,339 (35.2%) | 1,261 (35.0%) |  |
| 1959-1969 | 3,828 (36.8%) | 1,583 (40.8%) | 1,242 (33.2%) |  | 3,763 (36.5%) | 1,533 (40.3%) | 1,188 (33.0%) |  |
| *Region* |  |  |  | < 0.001 |  |  |  | < 0.001 |
| East | 3,472 (33.3%) | 1,549 (40.0%) | 1,155 (30.9%) |  | 3,437 (33.3%) | 1,519 (39.9%) | 1,114 (30.9%) |  |
| Middle | 3,225 (31.0%) | 1,047 (27.0%) | 1,512 (40.4%) |  | 3,186 (30.9%) | 1,030 (27.1%) | 1,458 (40.5%) |  |
| West | 3,716 (35.7%) | 1,280 (33.0%) | 1,076 (28.7%) |  | 3,690 (35.8%) | 1,258 (33.0%) | 1,032 (28.6%) |  |
| *Check-up use (binary)* |  |  |  | < 0.001 |  |  |  | < 0.001 |
| No | 8,406 (80.7%) | 2,950 (76.1%) | 2,192 (58.6%) |  | 7,784 (75.5%) | 2,647 (69.5%) | 1,518 (42.1%) |  |
| Yes | 2,007 (19.3%) | 926 (23.9%) | 1,551 (41.4%) |  | 2,529 (24.5%) | 1,160 (30.5%) | 2,086 (57.9%) |  |
| *Initial use (median)* | 2006 | 2006 | 1994 | < 0.001 | 2007 | 2005 | 1983 | < 0.001 |
| **Predictors** | **Breast Exam** | | |  | **Prostate cancer Exam** | | |  |
|  | **Rural residents with rural *hukou*** | **Urban residents with rural *hukou*** | **Urban *hukou* holders** |  | **Rural residents with rural *hukou*** | **Urban residents with rural *hukou*** | **Urban *hukou* holders** |  |
|  | (N = 5,440) | (N = 2,098) | (N = 1,769) | *p-value* | (N = 5,003) | (N = 1,814) | (N = 2,081) | *p-value* |
| *Education* |  |  |  | < 0.001 |  |  |  | < 0.001 |
| Illiterate | 3,154 (58.0%) | 924 (44.0%) | 305 (17.2%) |  | 1,123 (22.4%) | 305 (16.8%) | 128 (6.2%) |  |
| Less than middle school | 1,322 (24.3%) | 583 (27.8%) | 378 (21.4%) |  | 1,778 (35.5%) | 659 (36.3%) | 440 (21.1%) |  |
| Middle school | 790 (14.5%) | 460 (21.9%) | 534 (30.2%) |  | 1,545 (30.9%) | 612 (33.7%) | 636 (30.6%) |  |
| High School | 171 (3.1%) | 120 (5.7%) | 423 (23.9%) |  | 533 (10.7%) | 215 (11.9%) | 583 (28.0%) |  |
| College and above | 3 (0.1%) | 11 (0.5%) | 129 (7.3%) |  | 24 (0.5%) | 23 (1.3%) | 294 (14.1%) |  |
| *Marital status* |  |  |  | 0.523 |  |  |  | 0.002 |
| Continuously married | 4,246 (78.1%) | 1,634 (77.9%) | 1,358 (76.8%) |  | 4,191 (83.8%) | 1,570 (86.5%) | 1,796 (86.3%) |  |
| Ever disrupted or never married | 1,194 (21.9%) | 464 (22.1%) | 411 (23.2%) |  | 812 (16.2%) | 244 (13.5%) | 285 (13.7%) |  |
| *Gender* |  |  |  | / |  |  |  | / |
| Male | / | / | / |  | 5,003 (100.0%) | 1,814 (100.0%) | 2,081 (100.0%) |  |
| Female | 5,440 (100.0%) | 2,098 (100.0%) | 1,769 (100.0%) |  | / | / | / |  |
| *Main work* |  |  |  | < 0.001 |  |  |  | < 0.001 |
| Non-agricultural labor | 222 (4.1%) | 298 (14.2%) | 883 (49.9%) |  | 998 (19.9%) | 660 (36.4%) | 1,216 (58.4%) |  |
| Economic inactivity | 622 (11.4%) | 286 (13.6%) | 346 (19.6%) |  | 370 (7.4%) | 133 (7.3%) | 105 (5.0%) |  |
| Agricultural labor | 4,596 (84.5%) | 1,514 (72.2%) | 540 (30.5%) |  | 3,635 (72.7%) | 1,021 (56.3%) | 760 (36.5%) |  |
| *Hospitalization in adulthood* |  |  |  | 0.001 |  |  |  | < 0.001 |
| No | 4,866 (89.4%) | 1,906 (90.8%) | 1,543 (87.2%) |  | 4,301 (86.0%) | 1,559 (85.9%) | 1,665 (80.0%) |  |
| At least once | 574 (10.6%) | 192 (9.2%) | 226 (12.8%) |  | 702 (14.0%) | 255 (14.1%) | 416 (20.0%) |  |
| *Birth Group* |  |  |  | 0.060 |  |  |  | < 0.001 |
| 1917-1948 | 1,476 (27.1%) | 519 (24.7%) | 487 (27.5%) |  | 1,420 (28.4%) | 439 (24.2%) | 730 (35.1%) |  |
| 1949-1958 | 1,902 (35.0%) | 739 (35.2%) | 646 (36.5%) |  | 1,810 (36.2%) | 628 (34.6%) | 707 (34.0%) |  |
| 1959-1969 | 2,062 (37.9%) | 840 (40.0%) | 636 (36.0%) |  | 1,773 (35.4%) | 747 (41.2%) | 644 (30.9%) |  |
| *Region* |  |  |  | < 0.001 |  |  |  | < 0.001 |
| East | 1,830 (33.6%) | 820 (39.1%) | 559 (31.6%) |  | 1,667 (33.3%) | 746 (41.1%) | 633 (30.4%) |  |
| Middle | 1,676 (30.8%) | 590 (28.1%) | 704 (39.8%) |  | 1,553 (31.0%) | 472 (26.0%) | 848 (40.7%) |  |
| West | 1,934 (35.6%) | 688 (32.8%) | 506 (28.6%) |  | 1,783 (35.6%) | 596 (32.9%) | 600 (28.8%) |  |
| *Check-up use (binary)* |  |  |  | < 0.001 |  |  |  | < 0.001 |
| No | 4,562 (83.9%) | 1,701 (81.1%) | 1,074 (60.7%) |  | 4,614 (92.2%) | 1,663 (91.7%) | 1,763 (84.7%) |  |
| Yes | 878 (16.1%) | 397 (18.9%) | 695 (39.3%) |  | 389 (7.8%) | 151 (8.3%) | 318 (15.3%) |  |
| *Initial use (median)* | 2010 | 2009 | 2000 | < 0.001 | 2010 | 2010 | 2008 | < 0.001 |

*Note: Significance tests for categorical variables were performed using the Chi-square test. To compare urban-rural differences in median values of initial health check-up timing, we employed the Kruskal-Wallis test.*

Table A2. AFT Models for Duration of Being without Health Checkups (Rural residents with rural *hukou* as the reference group)

|  | Blood Pressure Test | | | Blood Test | | | Breast Exam | | | Prostate Cancer Exam | | | Dental Exam | | | Vision Exam | | |
| --- | --- | --- | --- | --- | --- | --- | --- | --- | --- | --- | --- | --- | --- | --- | --- | --- | --- | --- |
| Predictors | TR | 95% CI |  | TR | 95% CI |  | TR | 95% CI |  | TR | 95% CI |  | TR | 95% CI |  | TR | 95% CI |  |
| **Urban-rural (Ref = Rural residents with rural *hukou*)** | | | | | | | | | | | | | | | | | | |
| Urban residents with rural *hukou* | 0.988 | [0.975, 1.001] |  | 0.973 | [0.960, 0.986] | *** | 1.002 | [0.975, 1.029] |  | 0.986 | [0.951, 1.022] |  | 0.941 | [0.914, 0.969] | *** | 0.923 | [0.893, 0.954] | *** |
| Urban *hukou* holder | 0.883 | [0.870, 0.896] | *** | 0.894 | [0.881, 0.908] | *** | 0.893 | [0.868, 0.920] | *** | 0.919 | [0.888, 0.952] | *** | 0.798 | [0.774, 0.823] | *** | 0.722 | [0.699, 0.747] | *** |
| Education (Ref = Illiterate) |  |  |  |  |  |  |  |  |  |  |  |  |  |  |  |  |  |  |
| Less than Middle school | 0.934 | [0.921, 0.947] | *** | 0.935 | [0.921, 0.948] | *** | 0.940 | [0.914, 0.968] | *** | 0.954 | [0.917, 0.992] | * | 0.890 | [0.862, 0.919] | *** | 0.838 | [0.807, 0.869] | *** |
| Middle school | 0.888 | [0.874, 0.902] | *** | 0.896 | [0.881, 0.910] | *** | 0.865 | [0.839, 0.891] | *** | 0.961 | [0.920, 1.004] |  | 0.851 | [0.822, 0.881] | *** | 0.728 | [0.699, 0.757] | *** |
| High school | 0.834 | [0.818, 0.850] | *** | 0.858 | [0.841, 0.875] | *** | 0.800 | [0.772, 0.830] | *** | 0.950 | [0.904, 0.998] | * | 0.767 | [0.736, 0.798] | *** | 0.582 | [0.556, 0.609] | *** |
| College and above | 0.702 | [0.680, 0.724] | *** | 0.721 | [0.699, 0.743] | *** | 0.639 | [0.607, 0.673] | *** | 0.843 | [0.793, 0.895] | *** | 0.636 | [0.601, 0.674] | *** | 0.423 | [0.397, 0.451] | *** |
| Marital (Ref = Continuously married) |  |  |  |  |  |  |  |  |  |  |  |  |  |  |  |  |  |  |
| Ever disrupted or never married | 1.030 | [1.016, 1.044] | *** | 1.041 | [1.027, 1.056] | *** | 1.073 | [1.043, 1.104] | *** | 1.046 | [1.009, 1.085] | * | 1.037 | [1.007, 1.068] | * | 0.992 | [0.960, 1.025] |  |
| Gender (ref = Male) |  |  |  |  |  |  |  |  |  |  |  |  |  |  |  |  |  |  |
| Female | 0.995 | [0.984, 1.006] |  | 0.979 | [0.968, 0.990] | *** | / | / | / | / | / | / | 0.946 | [0.924, 0.968] | *** | 1.171 | [1.140, 1.202] | *** |
| Main work (Ref = Non-agricultural) |  |  |  |  |  |  |  |  |  |  |  |  |  |  |  |  |  |  |
| Economic inactivity | 1.058 | [1.038, 1.080] | *** | 1.068 | [1.046, 1.090] | *** | 1.113 | [1.075, 1.151] | *** | 1.055 | [0.993, 1.121] |  | 1.096 | [1.051, 1.143] | *** | 1.172 | [1.118, 1.228] | *** |
| Agricultural labor | 1.037 | [1.023, 1.051] | *** | 1.049 | [1.035, 1.064] | *** | 1.099 | [1.069, 1.129] | *** | 1.007 | [0.977, 1.038] |  | 1.064 | [1.035, 1.094] | *** | 1.141 | [1.107, 1.176] | *** |
| Hospitalization in adulthood (Ref = No) |  |  |  |  |  |  |  |  |  |  |  |  |  |  |  |  |  |  |
| At least once | 0.930 | [0.917, 0.944] | *** | 0.895 | [0.882, 0.907] | *** | 0.943 | [0.915, 0.972] | *** | 0.933 | [0.905, 0.962] | *** | 0.912 | [0.885, 0.940] | *** | 0.880 | [0.851, 0.909] | *** |
| Birth Group (Ref = 1917-1948) |  |  |  |  |  |  |  |  |  |  |  |  |  |  |  |  |  |  |
| Birth cohort: 1949-1958 | 0.850 | [0.839, 0.861] | *** | 0.856 | [0.844, 0.867] | *** | 0.782 | [0.759, 0.807] | *** | 0.900 | [0.872, 0.928] | *** | 0.859 | [0.836, 0.884] | *** | 0.906 | [0.878, 0.934] | *** |
| Birth cohort: 1959-1969 | 0.728 | [0.718, 0.739] | *** | 0.736 | [0.725, 0.747] | *** | 0.613 | [0.594, 0.632] | *** | 0.794 | [0.765, 0.823] | *** | 0.787 | [0.763, 0.811] | *** | 0.850 | [0.822, 0.880] | *** |
| Region (Ref = East) |  |  |  |  |  |  |  |  |  |  |  |  |  |  |  |  |  |  |
| Middle | 1.029 | [1.016, 1.042] | *** | 1.016 | [1.003, 1.028] | * | 1.090 | [1.064, 1.116] | *** | 1.004 | [0.973, 1.037] |  | 1.102 | [1.073, 1.131] | *** | 1.110 | [1.078, 1.144] | *** |
| West | 1.038 | [1.025, 1.051] | *** | 1.017 | [1.005, 1.031] | ** | 1.061 | [1.036, 1.087] | *** | 0.985 | [0.955, 1.016] |  | 1.093 | [1.064, 1.123] | *** | 1.051 | [1.021, 1.083] | *** |
| Intercept | 73.003 | [71.441, 74.598] | *** | 79.896 | [78.136, 81.695] | *** | 106.447 | [101.727, 111.387] | *** | 112.087 | [105.570, 119.006] | *** | 116.568 | [111.100, 122.305] | *** | 106.654 | [101.171, 112.434] | *** |
| Log(scale) | 0.275 | [0.271, 0.280] | *** | 0.256 | [0.252, 0.261] | *** | 0.221 | [0.212, 0.230] | *** | 0.189 | [0.177, 0.201] | *** | 0.369 | [0.359, 0.380] | *** | 0.469 | [0.458, 0.480] | *** |
| AIC | 104898.7 | | | 91149.2 | | | 21756.4 | | | 11073.0  8,898 | | | 53008.3 | | | 64937.3 | | |
| Sample size | 16,374 | | | 17,214 | | | 9,307 | | |  |  |  | 18,032 | | | 17,724 | | |

*Note. * p<0.05; ** p<0.01; *** p<0.001. The results are derived from Accelerated Failure Time (AFT) Weibull models. TR is the Time ratio (exp(β)) from AFT models. The reported coefficients for Log(scale) are exp(log(scale)), representing the scales of AFT models.*

Table A3. AICs for Accelerated Failure Time Models Across Outcome Distributions

| Outcome | Weibull | Log-normal | Log-logistic | Exponential | **Best Model** |
| --- | --- | --- | --- | --- | --- |
| Blood pressure | **104898.7** | 106621.4 | 105939.8 | 120395.9 | Weibull |
| Blood test | **91149.2** | 92766.1 | 91862.2 | 104538.7 | Weibull |
| Dental | 53008.3 | 53016.6 | **52985.7** | 56575.6 | Log-logistic |
| Vision | 64937.3 | **64296.6** | 64549.3 | 68007.6 | Log-normal |
| Breast | 21756.4 | 21781.6 | **21709.7** | 24933.9 | Log-logistic |
| Prostate cancer | **11073.0** | 11254.7 | 11086.1 | 12623.3 | Weibull |

Table A4. Accelerated Failure Time Models with Non-Weibull Distributions for the Smallest AIC

|  | Breast | | | Dental | | | Vision | | |
| --- | --- | --- | --- | --- | --- | --- | --- | --- | --- |
|  | TR | 95% CI |  | TR | 95% CI |  | TR | 95% CI |  |
| Urban-rural (Ref = Urban residents with rural *hukou*) | | | | | | | | | |
| Rural residents with rural *hukou* | 0.998 | [0.971, 1.025] |  | 1.059 | [1.027, 1.091] | *** | 1.075 | [1.038, 1.113] | *** |
| Urban *hukou* holder | 0.884 | [0.855, 0.914] | *** | 0.820 | [0.791, 0.851] | *** | 0.736 | [0.705, 0.767] | *** |
| Education (Ref = Illiterate) |  |  |  |  |  |  |  |  |  |
| Less than Middle school | 0.943 | [0.916, 0.970] | *** | 0.886 | [0.858, 0.916] | *** | 0.819 | [0.788, 0.850] | *** |
| Middle school | 0.862 | [0.837, 0.889] | *** | 0.843 | [0.813, 0.874] | *** | 0.683 | [0.656, 0.712] | *** |
| High school | 0.780 | [0.750, 0.811] | *** | 0.745 | [0.713, 0.779] | *** | 0.512 | [0.487, 0.539] | *** |
| College and above | 0.591 | [0.553, 0.632] | *** | 0.564 | [0.525, 0.605] | *** | 0.366 | [0.338, 0.398] | *** |
| Marital (Ref = Continuously married) |  |  |  |  |  |  |  |  |  |
| Ever disrupted or never married | 1.077 | [1.047, 1.109] | *** | 1.039 | [1.007, 1.072] | * | 0.998 | [0.964, 1.035] |  |
| Gender (Ref = Male) |  |  |  |  |  |  |  |  |  |
| Female | / | / | / | 0.953 | [0.929, 0.977] | *** | 1.300 | [1.263, 1.338] | *** |
| Main work (Ref = Non-agricultural) |  |  |  |  |  |  |  |  |  |
| Economic inactivity | 1.131 | [1.090, 1.173] | *** | 1.108 | [1.059, 1.159] | *** | 1.197 | [1.137, 1.260] | *** |
| Agricultural labor | 1.123 | [1.090, 1.157] | *** | 1.076 | [1.043, 1.110] | *** | 1.177 | [1.137, 1.218] | *** |
| Hospitalization in adulthood (Ref = No) |  |  |  |  |  |  |  |  |  |
| At least once | 0.935 | [0.905, 0.965] | *** | 0.896 | [0.867, 0.926] | *** | 0.862 | [0.831, 0.895] | *** |
| Birth Group (Ref = 1917-1948) |  |  |  |  |  |  |  |  |  |
| Birth cohort: 1949-1958 | 0.789 | [0.765, 0.814] | *** | 0.864 | [0.838, 0.890] | *** | 0.908 | [0.878, 0.940] | *** |
| Birth cohort: 1959-1969 | 0.619 | [0.599, 0.638] | *** | 0.797 | [0.771, 0.824] | *** | 0.892 | [0.859, 0.926] | *** |
| Region (Ref = East) |  |  |  |  |  |  |  |  |  |
| Middle | 1.089 | [1.062, 1.117] | *** | 1.113 | [1.082, 1.145] | *** | 1.108 | [1.072, 1.144] | *** |
| West | 1.062 | [1.035, 1.089] | *** | 1.107 | [1.075, 1.139] | *** | 1.058 | [1.024, 1.092] | *** |
| Intercept | 98.027 | [93.611, 102.651] | *** | 106.260 | [101.003, 111.791] | *** | 90.261 | [85.253, 95.563] | *** |
| Log(scale) | 0.197 | [0.189, 0.205] | *** | 0.340 | [0.331, 0.349] | *** | 0.714 | [0.700, 0.729] | *** |
| Sample size | 9,307 | | | 18,032 | | | 17,724 | | |

*Note. TR is the Time ratio (exp(β)) from AFT models. * p<0.05; ** p<0.01; *** p<0.001. The reported coefficients for Log(scale) are exp(log(scale)), representing the scales of AFT models. The 95% CIs are similarly exponentiated.*

Figure A2. KM Curves for Duration of Being Without Health Checkups (by Age, Cohort: 1917-1948)

Figure A3. KM Curves for Duration of Being Without Health Checkups (by Age, Cohort: 1949-1958)

Figure A4. KM Curves for Duration of Being Without Health Checkups (by Age, Cohort: 1959-1969)

Table A5. AFT models for initial health check-up utilizations (Cohort: 1917-1948)

|  | Blood Pressure Test | | | Blood Test | | | Breast Exam | | | Prostate Cancer Exam | | | Dental Exam | | | Vision Exam | | |
| --- | --- | --- | --- | --- | --- | --- | --- | --- | --- | --- | --- | --- | --- | --- | --- | --- | --- | --- |
| Predictors | TR | 95% CI |  | TR | 95% CI |  | TR | 95% CI |  | TR | 95% CI |  | TR | 95% CI |  | TR | 95% CI |  |
| Urban-rural (Ref = Urban residents with rural *hukou*) | | | | | | | | | | | | | | | | | | |
| Rural residents with rural *hukou* | 1.002 | [0.978, 1.028] |  | 1.018 | [0.993, 1.043] |  | 1.036 | [0.969, 1.108] |  | 1.018 | [0.968, 1.071] |  | 1.060 | [1.001, 1.124] | * | 1.132 | [1.069, 1.198] | *** |
| Urban *hukou* holder | 0.900 | [0.872, 0.928] | *** | 0.918 | [0.891, 0.946] | *** | 0.937 | [0.866, 1.013] |  | 0.957 | [0.905, 1.013] |  | 0.813 | [0.762, 0.869] | *** | 0.808 | [0.759, 0.861] | *** |
| Education (Ref = Illiterate) |  |  |  |  |  |  |  |  |  |  |  |  |  |  |  |  |  |  |
| Less than Middle school | 0.910 | [0.890, 0.931] | *** | 0.920 | [0.900, 0.941] | *** | 0.991 | [0.928, 1.058] |  | 0.950 | [0.906, 0.995] | * | 0.865 | [0.821, 0.913] | *** | 0.841 | [0.798, 0.886] | *** |
| Middle school | 0.858 | [0.832, 0.885] | *** | 0.868 | [0.842, 0.894] | *** | 0.869 | [0.799, 0.946] | ** | 0.909 | [0.860, 0.962] | *** | 0.798 | [0.745, 0.855] | *** | 0.733 | [0.685, 0.784] | *** |
| High school | 0.842 | [0.808, 0.877] | *** | 0.868 | [0.834, 0.903] | *** | 0.727 | [0.660, 0.799] | *** | 0.989 | [0.917, 1.066] |  | 0.736 | [0.677, 0.799] | *** | 0.637 | [0.587, 0.690] | *** |
| College and above | 0.743 | [0.700, 0.788] | *** | 0.787 | [0.744, 0.832] | *** | 0.736 | [0.635, 0.855] | *** | 0.923 | [0.845, 1.008] |  | 0.653 | [0.585, 0.729] | *** | 0.465 | [0.418, 0.518] | *** |
| Marital (Ref = Continuously married) |  |  |  |  |  |  |  |  |  |  |  |  |  |  |  |  |  |  |
| Ever disrupted or never married | 1.055 | [1.034, 1.076] | *** | 1.063 | [1.042, 1.084] | *** | 1.151 | [1.092, 1.212] | *** | 1.073 | [1.029, 1.120] | ** | 1.060 | [1.014, 1.109] | * | 1.043 | [0.997, 1.090] |  |
| Gender (ref = Male) |  |  |  |  |  |  |  |  |  |  |  |  |  |  |  |  |  |  |
| Female | 0.972 | [0.953, 0.992] | ** | 0.974 | [0.954, 0.993] | ** | / | / | / | / | / | / | 0.954 | [0.912, 0.998] | * | 1.047 | [1.001, 1.095] | * |
| Main work (Ref = Non-agricultural) |  |  |  |  |  |  |  |  |  |  |  |  |  |  |  |  |  |  |
| Economic inactivity | 1.111 | [1.069, 1.155] | *** | 1.103 | [1.062, 1.145] | *** | 1.165 | [1.060, 1.281] | ** | 1.012 | [0.938, 1.091] |  | 1.123 | [1.032, 1.222] | ** | 1.189 | [1.094, 1.291] | *** |
| Agricultural labor | 1.042 | [1.014, 1.070] | ** | 1.031 | [1.004, 1.058] | * | 1.103 | [1.025, 1.186] | ** | 1.001 | [0.957, 1.046] |  | 1.017 | [0.962, 1.074] |  | 1.078 | [1.022, 1.138] | ** |
| Hospitalization in adulthood (Ref = No) |  |  |  |  |  |  |  |  |  |  |  |  |  |  |  |  |  |  |
| At least once | 0.930 | [0.909, 0.953] | *** | 0.908 | [0.888, 0.929] | *** | 0.935 | [0.878, 0.996] | * | 0.941 | [0.903, 0.980] | ** | 0.911 | [0.866, 0.959] | *** | 0.867 | [0.825, 0.910] | *** |
| Region (Ref = East) |  |  |  |  |  |  |  |  |  |  |  |  |  |  |  |  |  |  |
| Middle | 1.019 | [0.996, 1.042] |  | 1.017 | [0.995, 1.039] |  | 1.007 | [0.949, 1.069] |  | 0.985 | [0.944, 1.028] |  | 1.114 | [1.059, 1.172] | *** | 1.074 | [1.022, 1.128] | ** |
| West | 1.029 | [1.006, 1.052] | * | 1.019 | [0.997, 1.041] |  | 0.998 | [0.940, 1.059] |  | 0.986 | [0.945, 1.029] |  | 1.034 | [0.984, 1.085] |  | 1.001 | [0.953, 1.050] |  |
| Intercept | 74.092 | [71.269, 77.026] | *** | 80.587 | [77.583, 83.707] | *** | 103.611 | [93.631, 114.654] | *** | 107.501 | [99.465, 116.187] | *** | 126.541 | [115.715, 138.380] | *** | 108.923 | [100.103, 118.520] | *** |
| Log(scale) | 0.268 | [0.260, 0.276] | *** | 0.242 | [0.234, 0.250] | *** | 0.227 | [0.206, 0.251] | *** | 0.169 | [0.154, 0.185] | *** | 0.380 | [0.361, 0.399] | *** | 0.438 | [0.420, 0.457] | *** |
| AIC | 31510.0 | | | 27835.8 | | | 4240.0 | | | 4534.2  2,589 | | | 16260.9 | | | 20998.6 | | |
| Sample size | 4,503 | | | 4,735 | | | 2,482 | | |  |  |  | 5,017 | | | 4,966 | | |

*Note. * p<0.05; ** p<0.01; *** p<0.001. The results are derived from Accelerated Failure Time (AFT) Weibull models. TR is the Time ratio (exp(β)) from AFT models. The reported coefficients for Log(scale) are exp(log(scale)), representing the scales of AFT models.*

Table A6. AFT models for initial health check-up utilizations (Cohort: 1949-1958)

|  | Blood Pressure Test | | | Blood Test | | | Breast Exam | | | Prostate Cancer Exam | | | Dental Exam | | | Vision Exam | | |
| --- | --- | --- | --- | --- | --- | --- | --- | --- | --- | --- | --- | --- | --- | --- | --- | --- | --- | --- |
| Predictors | TR | 95% CI |  | TR | 95% CI |  | TR | 95% CI |  | TR | 95% CI |  | TR | 95% CI |  | TR | 95% CI |  |
| Urban-rural (Ref = Urban residents with rural *hukou*) | | | | | | | | | | | | | | | | | | |
| Rural residents with rural *hukou* | 1.031 | [1.008, 1.054] | ** | 1.027 | [1.004, 1.051] | * | 0.980 | [0.928, 1.035] |  | 1.008 | [0.942, 1.078] |  | 1.051 | [1.001, 1.104] | * | 1.068 | [1.006, 1.134] | * |
| Urban *hukou* holder | 0.891 | [0.866, 0.917] | *** | 0.911 | [0.886, 0.937] | *** | 0.858 | [0.805, 0.915] | *** | 0.925 | [0.856, 1.000] | * | 0.827 | [0.781, 0.876] | *** | 0.738 | [0.690, 0.791] | *** |
| Education (Ref = Illiterate) |  |  |  |  |  |  |  |  |  |  |  |  |  |  |  |  |  |  |
| Less than Middle school | 0.961 | [0.940, 0.984] | *** | 0.951 | [0.929, 0.974] | *** | 0.935 | [0.888, 0.985] | * | 0.972 | [0.901, 1.048] |  | 0.897 | [0.853, 0.944] | *** | 0.798 | [0.748, 0.852] | *** |
| Middle school | 0.912 | [0.888, 0.935] | *** | 0.897 | [0.873, 0.920] | *** | 0.876 | [0.824, 0.931] | *** | 0.971 | [0.897, 1.051] |  | 0.864 | [0.817, 0.914] | *** | 0.700 | [0.653, 0.751] | *** |
| High school | 0.832 | [0.806, 0.858] | *** | 0.848 | [0.822, 0.876] | *** | 0.755 | [0.706, 0.807] | *** | 0.938 | [0.857, 1.027] |  | 0.801 | [0.750, 0.855] | *** | 0.563 | [0.521, 0.610] | *** |
| College and above | 0.699 | [0.656, 0.744] | *** | 0.710 | [0.670, 0.753] | *** | 0.569 | [0.512, 0.633] | *** | 0.803 | [0.713, 0.904] | *** | 0.663 | [0.595, 0.739] | *** | 0.446 | [0.391, 0.508] | *** |
| Marital (Ref = Continuously married) |  |  |  |  |  |  |  |  |  |  |  |  |  |  |  |  |  |  |
| Ever disrupted or never married | 1.007 | [0.982, 1.031] |  | 1.026 | [1.001, 1.052] | * | 1.050 | [0.997, 1.106] |  | 1.017 | [0.941, 1.100] |  | 1.032 | [0.979, 1.087] |  | 0.974 | [0.915, 1.037] |  |
| Gender (ref = Male) |  |  |  |  |  |  |  |  |  |  |  |  |  |  |  |  |  |  |
| Female | 1.016 | [0.997, 1.035] |  | 0.973 | [0.954, 0.991] | ** | / | / | / | / | / | / | 0.959 | [0.922, 0.997] | * | 1.192 | [1.136, 1.251] | *** |
| Main work (Ref = Non-agricultural) |  |  |  |  |  |  |  |  |  |  |  |  |  |  |  |  |  |  |
| Economic inactivity | 1.041 | [1.004, 1.080] | * | 1.043 | [1.006, 1.082] | * | 1.188 | [1.101, 1.282] | *** | 1.138 | [0.979, 1.322] |  | 1.106 | [1.025, 1.194] | ** | 1.121 | [1.026, 1.225] | * |
| Agricultural labor | 1.031 | [1.006, 1.057] | * | 1.044 | [1.018, 1.070] | *** | 1.129 | [1.067, 1.194] | *** | 1.007 | [0.950, 1.067] |  | 1.068 | [1.017, 1.122] | ** | 1.159 | [1.095, 1.227] | *** |
| Hospitalization in adulthood (Ref = No) |  |  |  |  |  |  |  |  |  |  |  |  |  |  |  |  |  |  |
| At least once | 0.924 | [0.901, 0.948] | *** | 0.890 | [0.869, 0.912] | *** | 0.938 | [0.887, 0.991] | * | 0.906 | [0.854, 0.960] | *** | 0.937 | [0.891, 0.987] | * | 0.900 | [0.847, 0.957] | *** |
| Region (Ref = East) |  |  |  |  |  |  |  |  |  |  |  |  |  |  |  |  |  |  |
| Middle | 1.037 | [1.016, 1.060] | *** | 1.029 | [1.008, 1.051] | ** | 1.138 | [1.086, 1.194] | *** | 1.017 | [0.959, 1.078] |  | 1.110 | [1.062, 1.160] | *** | 1.105 | [1.048, 1.165] | *** |
| West | 1.039 | [1.017, 1.062] | *** | 1.027 | [1.006, 1.050] | * | 1.076 | [1.028, 1.126] | ** | 1.017 | [0.958, 1.079] |  | 1.116 | [1.067, 1.167] | *** | 1.077 | [1.020, 1.138] | ** |
| Intercept | 60.350 | [58.313, 62.459] | *** | 68.557 | [66.186, 71.014] | *** | 85.055 | [78.765, 91.848] | *** | 103.850 | [92.617, 116.446] | *** | 98.445 | [91.262, 106.193] | *** | 102.363 | [93.523, 112.040] | *** |
| Log(scale) | 0.281 | [0.273, 0.289] | *** | 0.256 | [0.248, 0.264] | *** | 0.237 | [0.220, 0.255] | *** | 0.204 | [0.182, 0.229] | *** | 0.373 | [0.356, 0.391] | *** | 0.497 | [0.478, 0.518] | *** |
| AIC | 37545.9 | | | 32306.9 | | | 7062.3 | | | 3737.8  3,145 | | | 19351.2 | | | 22747.8 | | |
| Sample size | 5,866 | | | 6,132 | | | 3,287 | | |  |  |  | 6,362 | | | 6,274 | | |

*Note. * p<0.05; ** p<0.01; *** p<0.001. The results are derived from Accelerated Failure Time (AFT) Weibull models. TR is the Time ratio (exp(β)) from AFT models. The reported coefficients for Log(scale) are exp(log(scale)), representing the scales of AFT models.*

Table A7. AFT models for initial health check-up utilizations (Cohort: 1959-1969)

|  | Blood Pressure Test | | | Blood Test | | | Breast Exam | | | Prostate Cancer Exam | | | Dental Exam | | | Vision Exam | | |
| --- | --- | --- | --- | --- | --- | --- | --- | --- | --- | --- | --- | --- | --- | --- | --- | --- | --- | --- |
| Predictors | TR | 95% CI |  | TR | 95% CI |  | TR | 95% CI |  | TR | 95% CI |  | TR | 95% CI |  | TR | 95% CI |  |
| Urban-rural (Ref = Urban residents with rural *hukou*) | | | | | | | | | | | | | | | | | | |
| Rural residents with rural *hukou* | 1.003 | [0.982, 1.024] |  | 1.035 | [1.012, 1.059] | ** | 0.998 | [0.965, 1.033] |  | 1.021 | [0.947, 1.102] |  | 1.074 | [1.025, 1.125] | ** | 1.057 | [1.001, 1.117] | * |
| Urban *hukou* holder | 0.893 | [0.869, 0.917] | *** | 0.929 | [0.903, 0.956] | *** | 0.901 | [0.866, 0.937] | *** | 0.906 | [0.831, 0.987] | * | 0.915 | [0.867, 0.967] | ** | 0.799 | [0.750, 0.852] | *** |
| Education (Ref = Illiterate) |  |  |  |  |  |  |  |  |  |  |  |  |  |  |  |  |  |  |
| Less than Middle school | 0.931 | [0.905, 0.958] | *** | 0.937 | [0.908, 0.967] | *** | 0.927 | [0.889, 0.967] | *** | 0.921 | [0.810, 1.047] |  | 0.945 | [0.885, 1.008] |  | 0.852 | [0.780, 0.932] | *** |
| Middle school | 0.886 | [0.863, 0.909] | *** | 0.910 | [0.884, 0.937] | *** | 0.870 | [0.837, 0.905] | *** | 0.985 | [0.871, 1.113] |  | 0.901 | [0.849, 0.957] | *** | 0.721 | [0.664, 0.782] | *** |
| High school | 0.835 | [0.809, 0.862] | *** | 0.863 | [0.834, 0.894] | *** | 0.840 | [0.801, 0.881] | *** | 0.906 | [0.797, 1.029] |  | 0.787 | [0.735, 0.842] | *** | 0.549 | [0.502, 0.600] | *** |
| College and above | 0.676 | [0.645, 0.710] | *** | 0.684 | [0.651, 0.718] | *** | 0.648 | [0.608, 0.692] | *** | 0.771 | [0.667, 0.891] | *** | 0.617 | [0.564, 0.676] | *** | 0.371 | [0.332, 0.416] | *** |
| Marital (Ref = Continuously married) |  |  |  |  |  |  |  |  |  |  |  |  |  |  |  |  |  |  |
| Ever disrupted or never married | 1.008 | [0.980, 1.038] |  | 1.008 | [0.976, 1.040] |  | 1.025 | [0.982, 1.070] |  | 0.962 | [0.880, 1.051] |  | 0.976 | [0.919, 1.037] |  | 0.960 | [0.895, 1.030] |  |
| Gender (ref = Male) |  |  |  |  |  |  |  |  |  |  |  |  |  |  |  |  |  |  |
| Female | 0.991 | [0.974, 1.009] |  | 0.986 | [0.967, 1.006] |  | / | / | / | / | / | / | 0.916 | [0.881, 0.951] | *** | 1.272 | [1.215, 1.331] | *** |
| Main work (Ref = Non-agricultural) |  |  |  |  |  |  |  |  |  |  |  |  |  |  |  |  |  |  |
| Economic inactivity | 1.037 | [1.007, 1.069] | * | 1.053 | [1.020, 1.088] | ** | 1.068 | [1.026, 1.112] | ** | 1.096 | [0.965, 1.246] |  | 1.065 | [1.001, 1.132] | * | 1.170 | [1.086, 1.261] | *** |
| Agricultural labor | 1.040 | [1.019, 1.062] | *** | 1.066 | [1.042, 1.090] | *** | 1.088 | [1.053, 1.125] | *** | 1.025 | [0.965, 1.088] |  | 1.105 | [1.058, 1.154] | *** | 1.152 | [1.097, 1.210] | *** |
| Hospitalization in adulthood (Ref = No) |  |  |  |  |  |  |  |  |  |  |  |  |  |  |  |  |  |  |
| At least once | 0.932 | [0.908, 0.957] | *** | 0.880 | [0.856, 0.904] | *** | 0.958 | [0.917, 1.001] |  | 0.947 | [0.882, 1.017] |  | 0.880 | [0.836, 0.927] | *** | 0.875 | [0.823, 0.930] | *** |
| Region (Ref = East) |  |  |  |  |  |  |  |  |  |  |  |  |  |  |  |  |  |  |
| Middle | 1.030 | [1.009, 1.052] | ** | 1.002 | [0.980, 1.025] |  | 1.087 | [1.054, 1.121] | *** | 1.033 | [0.962, 1.110] |  | 1.075 | [1.029, 1.122] | ** | 1.161 | [1.102, 1.222] | *** |
| West | 1.044 | [1.023, 1.066] | *** | 1.008 | [0.985, 1.031] |  | 1.070 | [1.037, 1.104] | *** | 0.946 | [0.885, 1.012] |  | 1.121 | [1.071, 1.172] | *** | 1.077 | [1.023, 1.134] | ** |
| Intercept | 53.693 | [51.865, 55.587] | *** | 58.503 | [56.309, 60.783] | *** | 64.186 | [61.002, 67.536] | *** | 95.116 | [81.071, 111.594] | *** | 85.316 | [78.828, 92.338] | *** | 88.236 | [79.879, 97.467] | *** |
| Log(scale) | 0.274 | [0.267, 0.282] | *** | 0.267 | [0.259, 0.275] | *** | 0.206 | [0.194, 0.217] | *** | 0.203 | [0.178, 0.231] | *** | 0.354 | [0.337, 0.371] | *** | 0.467 | [0.449, 0.486] | *** |
| AIC | 35830.2 | | | 30976.2 | | | 10430.8 | | | 2803.3  3,164 | | | 17393.1 | | | 21137.7 | | |
| Sample size | 6,005 | | | 6,347 | | | 3,538 | | |  |  |  | 6,653 | | | 6,484 | | |

*Note. * p<0.05; ** p<0.01; *** p<0.001. The results are derived from Accelerated Failure Time (AFT) Weibull models. TR is the Time ratio (exp(β)) from AFT models. The reported coefficients for Log(scale) are exp(log(scale)), representing the scales of AFT models.*

Figure A5. KM Curves for Duration of Being Without Health Checkups (by Age, East Region)

Figure A6. KM Curves for Duration of Being Without Health Checkups (by Age, Middle Region)

Figure A7. KM Curves for Duration of Being Without Health Checkups (by Age, West Region)

Table A8. AFT models for initial health check-up utilizations (East Region)

|  | Blood Pressure Test | | | Blood Test | | | Breast Exam | | | Prostate Cancer Exam | | | Dental Exam | | | Vision Exam | | |
| --- | --- | --- | --- | --- | --- | --- | --- | --- | --- | --- | --- | --- | --- | --- | --- | --- | --- | --- |
| Predictors | TR | 95% CI |  | TR | 95% CI |  | TR | 95% CI |  | TR | 95% CI |  | TR | 95% CI |  | TR | 95% CI |  |
| Urban-rural (Ref = Urban residents with rural *hukou*) | | | | | | | | | | | | | | | | | | |
| Rural residents with rural *hukou* | 1.010 | [0.989, 1.032] |  | 1.024 | [1.002, 1.046] | * | 0.984 | [0.945, 1.025] |  | 0.996 | [0.933, 1.064] |  | 1.055 | [1.008, 1.103] | * | 1.119 | [1.065, 1.177] | *** |
| Urban *hukou* holder | 0.881 | [0.857, 0.906] | *** | 0.912 | [0.888, 0.937] | *** | 0.861 | [0.821, 0.902] | *** | 0.900 | [0.837, 0.968] | ** | 0.837 | [0.794, 0.883] | *** | 0.760 | [0.718, 0.805] | *** |
| Education (Ref = Illiterate) |  |  |  |  |  |  |  |  |  |  |  |  |  |  |  |  |  |  |
| Less than Middle school | 0.921 | [0.900, 0.944] | *** | 0.937 | [0.914, 0.960] | *** | 0.932 | [0.890, 0.975] | ** | 0.897 | [0.822, 0.979] | * | 0.891 | [0.846, 0.937] | *** | 0.820 | [0.772, 0.871] | *** |
| Middle school | 0.876 | [0.853, 0.899] | *** | 0.893 | [0.869, 0.918] | *** | 0.836 | [0.798, 0.876] | *** | 0.905 | [0.825, 0.993] | * | 0.874 | [0.826, 0.925] | *** | 0.743 | [0.697, 0.793] | *** |
| High school | 0.816 | [0.790, 0.843] | *** | 0.843 | [0.816, 0.871] | *** | 0.798 | [0.754, 0.844] | *** | 0.855 | [0.775, 0.944] | ** | 0.778 | [0.729, 0.831] | *** | 0.602 | [0.559, 0.648] | *** |
| College and above | 0.717 | [0.678, 0.758] | *** | 0.740 | [0.703, 0.780] | *** | 0.643 | [0.595, 0.696] | *** | 0.759 | [0.672, 0.856] | *** | 0.654 | [0.593, 0.720] | *** | 0.441 | [0.397, 0.490] | *** |
| Marital (Ref = Continuously married) |  |  |  |  |  |  |  |  |  |  |  |  |  |  |  |  |  |  |
| Ever disrupted or never married | 1.047 | [1.022, 1.073] | *** | 1.068 | [1.042, 1.094] | *** | 1.081 | [1.032, 1.132] | ** | 1.073 | [0.996, 1.156] |  | 1.029 | [0.980, 1.081] |  | 0.994 | [0.942, 1.049] |  |
| Gender (ref = Male) |  |  |  |  |  |  |  |  |  |  |  |  |  |  |  |  |  |  |
| Female | 0.983 | [0.964, 1.001] |  | 0.958 | [0.940, 0.976] | *** | / | / | / | / | / | / | 0.941 | [0.906, 0.978] | ** | 1.141 | [1.094, 1.191] | *** |
| Main work (Ref = Non-agricultural) |  |  |  |  |  |  |  |  |  |  |  |  |  |  |  |  |  |  |
| Economic inactivity | 1.077 | [1.042, 1.113] | *** | 1.105 | [1.069, 1.143] | *** | 1.155 | [1.094, 1.220] | *** | 1.201 | [1.045, 1.380] | ** | 1.110 | [1.037, 1.188] | ** | 1.183 | [1.099, 1.274] | *** |
| Agricultural labor | 1.030 | [1.007, 1.052] | ** | 1.055 | [1.032, 1.078] | *** | 1.098 | [1.055, 1.143] | *** | 1.033 | [0.980, 1.088] |  | 1.027 | [0.983, 1.073] |  | 1.096 | [1.046, 1.148] | *** |
| Hospitalization in adulthood (Ref = No) |  |  |  |  |  |  |  |  |  |  |  |  |  |  |  |  |  |  |
| At least once | 0.949 | [0.924, 0.974] | *** | 0.916 | [0.893, 0.940] | *** | 0.968 | [0.919, 1.019] |  | 0.937 | [0.883, 0.994] | * | 0.950 | [0.901, 1.001] |  | 0.927 | [0.876, 0.982] | ** |
| Birth Group (Ref = 1917-1948) |  |  |  |  |  |  |  |  |  |  |  |  |  |  |  |  |  |  |
| Birth cohort: 1949-1958 | 0.848 | [0.829, 0.867] | *** | 0.858 | [0.839, 0.877] | *** | 0.746 | [0.710, 0.784] | *** | 0.895 | [0.845, 0.948] | *** | 0.836 | [0.799, 0.875] | *** | 0.874 | [0.831, 0.919] | *** |
| Birth cohort: 1959-1969 | 0.727 | [0.710, 0.745] | *** | 0.750 | [0.732, 0.769] | *** | 0.590 | [0.561, 0.620] | *** | 0.819 | [0.764, 0.879] | *** | 0.760 | [0.722, 0.799] | *** | 0.796 | [0.753, 0.841] | *** |
| Intercept | 74.355 | [71.843, 76.954] | *** | 79.457 | [76.735, 82.275] | *** | 112.819 | [105.166, 121.028] | *** | 121.987 | [108.469, 137.189] | *** | 121.567 | [112.802, 131.014] | *** | 110.733 | [102.167, 120.017] | *** |
| Log(scale) | 0.278 | [0.270, 0.285] | *** | 0.255 | [0.247, 0.263] | *** | 0.222 | [0.208, 0.236] | *** | 0.202 | [0.181, 0.225] | *** | 0.376 | [0.360, 0.393] | *** | 0.461 | [0.444, 0.479] | *** |
| AIC | 36421.2 | | | 31701.1 | | | 8649.8 | | | 3696.8  3,046 | | | 20459.1 | | | 23915.3 | | |
| Sample size | 5,510 | | | 5,840 | | | 3,209 | | |  |  |  | 6,176 | | | 6,070 | | |

*Note. * p<0.05; ** p<0.01; *** p<0.001. The results are derived from Accelerated Failure Time (AFT) Weibull models. TR is the Time ratio (exp(β)) from AFT models. The reported coefficients for Log(scale) are exp(log(scale)), representing the scales of AFT models.*

Table A9. AFT models for initial health check-up utilizations (Middle Region)

|  | Blood Pressure Test | | | Blood Test | | | Breast Exam | | | Prostate Cancer Exam | | | Dental Exam | | | Vision Exam | | |
| --- | --- | --- | --- | --- | --- | --- | --- | --- | --- | --- | --- | --- | --- | --- | --- | --- | --- | --- |
| Predictors | TR | 95% CI |  | TR | 95% CI |  | TR | 95% CI |  | TR | 95% CI |  | TR | 95% CI |  | TR | 95% CI |  |
| Urban-rural (Ref = Urban residents with rural *hukou*) | | | | | | | | | | | | | | | | | | |
| Rural residents with rural *hukou* | 1.017 | [0.990, 1.044] |  | 1.024 | [0.997, 1.053] |  | 1.026 | [0.972, 1.083] |  | 1.044 | [0.978, 1.114] |  | 1.073 | [1.010, 1.140] | * | 1.117 | [1.045, 1.195] | ** |
| Urban *hukou* holder | 0.904 | [0.876, 0.932] | *** | 0.918 | [0.889, 0.947] | *** | 0.900 | [0.847, 0.955] | *** | 0.965 | [0.899, 1.036] |  | 0.829 | [0.777, 0.885] | *** | 0.816 | [0.760, 0.877] | *** |
| Education (Ref = Illiterate) |  |  |  |  |  |  |  |  |  |  |  |  |  |  |  |  |  |  |
| Less than Middle school | 0.936 | [0.911, 0.961] | *** | 0.927 | [0.901, 0.954] | *** | 0.919 | [0.868, 0.973] | ** | 0.988 | [0.922, 1.058] |  | 0.879 | [0.825, 0.937] | *** | 0.833 | [0.774, 0.897] | *** |
| Middle school | 0.895 | [0.870, 0.920] | *** | 0.890 | [0.864, 0.917] | *** | 0.864 | [0.815, 0.915] | *** | 0.977 | [0.907, 1.052] |  | 0.807 | [0.755, 0.862] | *** | 0.721 | [0.668, 0.779] | *** |
| High school | 0.849 | [0.820, 0.878] | *** | 0.861 | [0.830, 0.893] | *** | 0.790 | [0.737, 0.847] | *** | 0.996 | [0.914, 1.085] |  | 0.771 | [0.714, 0.832] | *** | 0.590 | [0.542, 0.643] | *** |
| College and above | 0.695 | [0.658, 0.734] | *** | 0.697 | [0.660, 0.736] | *** | 0.605 | [0.549, 0.666] | *** | 0.885 | [0.799, 0.979] | * | 0.630 | [0.568, 0.699] | *** | 0.414 | [0.369, 0.465] | *** |
| Marital (Ref = Continuously married) |  |  |  |  |  |  |  |  |  |  |  |  |  |  |  |  |  |  |
| Ever disrupted or never married | 1.033 | [1.006, 1.059] | * | 1.046 | [1.018, 1.075] | ** | 1.079 | [1.021, 1.142] | ** | 1.011 | [0.952, 1.074] |  | 1.052 | [0.994, 1.115] |  | 0.995 | [0.935, 1.059] |  |
| Gender (ref = Male) |  |  |  |  |  |  |  |  |  |  |  |  |  |  |  |  |  |  |
| Female | 1.022 | [1.002, 1.042] | * | 1.008 | [0.987, 1.029] |  | / | / | / | / | / | / | 0.967 | [0.926, 1.011] |  | 1.258 | [1.196, 1.324] | *** |
| Main work (Ref = Non-agricultural) |  |  |  |  |  |  |  |  |  |  |  |  |  |  |  |  |  |  |
| Economic inactivity | 1.031 | [0.995, 1.068] |  | 1.044 | [1.006, 1.084] | * | 1.009 | [0.949, 1.074] |  | 1.020 | [0.920, 1.131] |  | 1.073 | [0.994, 1.159] |  | 1.097 | [1.008, 1.195] | * |
| Agricultural labor | 1.014 | [0.989, 1.040] |  | 1.025 | [0.999, 1.052] |  | 1.016 | [0.963, 1.073] |  | 0.980 | [0.928, 1.035] |  | 1.059 | [1.005, 1.116] | * | 1.126 | [1.064, 1.193] | *** |
| Hospitalization in adulthood (Ref = No) |  |  |  |  |  |  |  |  |  |  |  |  |  |  |  |  |  |  |
| At least once | 0.920 | [0.896, 0.945] | *** | 0.859 | [0.837, 0.882] | *** | 0.920 | [0.869, 0.974] | ** | 0.922 | [0.875, 0.971] | ** | 0.876 | [0.830, 0.925] | *** | 0.880 | [0.827, 0.935] | *** |
| Birth Group (Ref = 1917-1948) |  |  |  |  |  |  |  |  |  |  |  |  |  |  |  |  |  |  |
| Birth cohort: 1949-1958 | 0.855 | [0.835, 0.876] | *** | 0.859 | [0.837, 0.880] | *** | 0.824 | [0.777, 0.874] | *** | 0.900 | [0.854, 0.949] | *** | 0.844 | [0.801, 0.890] | *** | 0.893 | [0.843, 0.946] | *** |
| Birth cohort: 1959-1969 | 0.725 | [0.706, 0.744] | *** | 0.729 | [0.709, 0.749] | *** | 0.631 | [0.595, 0.670] | *** | 0.810 | [0.758, 0.865] | *** | 0.761 | [0.718, 0.806] | *** | 0.870 | [0.815, 0.927] | *** |
| Intercept | 74.607 | [71.639, 77.699] | *** | 83.081 | [79.624, 86.688] | *** | 120.391 | [109.760, 132.053] | *** | 108.602 | [98.128, 120.195] | *** | 136.571 | [124.142, 150.244] | *** | 115.177 | [103.981, 127.578] | *** |
| Log(scale) | 0.288 | [0.280, 0.296] | *** | 0.275 | [0.266, 0.284] | *** | 0.229 | [0.212, 0.247] | *** | 0.187 | [0.168, 0.209] | *** | 0.387 | [0.368, 0.407] | *** | 0.493 | [0.472, 0.514] | *** |
| AIC | 33881.7 | | | 29851.3 | | | 6556.7 | | | 3590.9  2,873 | | | 16572.1 | | | 20475.3 | | |
| Sample size | 5,210 | | | 5,524 | | | 2,970 | | |  |  |  | 5,784 | | | 5,674 | | |

*Note. * p<0.05; ** p<0.01; *** p<0.001. The results are derived from Accelerated Failure Time (AFT) Weibull models. TR is the Time ratio (exp(β)) from AFT models. The reported coefficients for Log(scale) are exp(log(scale)), representing the scales of AFT models.*

Table A10. AFT models for initial health check-up utilizations (West Region)

|  | Blood Pressure Test | | | Blood Test | | | Breast Exam | | | Prostate Cancer Exam | | | Dental Exam | | | Vision Exam | | |
| --- | --- | --- | --- | --- | --- | --- | --- | --- | --- | --- | --- | --- | --- | --- | --- | --- | --- | --- |
| Predictors | TR | 95% CI |  | TR | 95% CI |  | TR | 95% CI |  | TR | 95% CI |  | TR | 95% CI |  | TR | 95% CI |  |
| Urban-rural (Ref = Urban residents with rural *hukou*) | | | | | | | | | | | | | | | | | | |
| Rural residents with rural *hukou* | 1.012 | [0.991, 1.035] |  | 1.035 | [1.013, 1.058] | ** | 0.996 | [0.951, 1.043] |  | 1.013 | [0.957, 1.072] |  | 1.068 | [1.017, 1.122] | ** | 1.020 | [0.962, 1.081] |  |
| Urban *hukou* holder | 0.891 | [0.867, 0.916] | *** | 0.924 | [0.899, 0.950] | *** | 0.935 | [0.885, 0.988] | * | 0.932 | [0.873, 0.997] | * | 0.888 | [0.838, 0.942] | *** | 0.778 | [0.726, 0.833] | *** |
| Education (Ref = Illiterate) |  |  |  |  |  |  |  |  |  |  |  |  |  |  |  |  |  |  |
| Less than Middle school | 0.944 | [0.924, 0.965] | *** | 0.940 | [0.919, 0.961] | *** | 0.969 | [0.923, 1.017] |  | 0.959 | [0.906, 1.015] |  | 0.902 | [0.857, 0.949] | *** | 0.864 | [0.813, 0.919] | *** |
| Middle school | 0.897 | [0.874, 0.919] | *** | 0.906 | [0.883, 0.930] | *** | 0.912 | [0.865, 0.961] | *** | 0.986 | [0.923, 1.052] |  | 0.871 | [0.821, 0.923] | *** | 0.718 | [0.671, 0.768] | *** |
| High school | 0.841 | [0.813, 0.869] | *** | 0.875 | [0.846, 0.905] | *** | 0.811 | [0.762, 0.864] | *** | 0.989 | [0.911, 1.073] |  | 0.750 | [0.700, 0.804] | *** | 0.551 | [0.509, 0.597] | *** |
| College and above | 0.706 | [0.668, 0.745] | *** | 0.737 | [0.700, 0.776] | *** | 0.679 | [0.613, 0.751] | *** | 0.872 | [0.793, 0.960] | ** | 0.624 | [0.564, 0.691] | *** | 0.411 | [0.367, 0.461] | *** |
| Marital (Ref = Continuously married) |  |  |  |  |  |  |  |  |  |  |  |  |  |  |  |  |  |  |
| Ever disrupted or never married | 1.013 | [0.991, 1.034] |  | 1.011 | [0.989, 1.034] |  | 1.059 | [1.010, 1.109] | * | 1.054 | [0.996, 1.114] |  | 1.035 | [0.988, 1.086] |  | 0.986 | [0.933, 1.041] |  |
| Gender (ref = Male) |  |  |  |  |  |  |  |  |  |  |  |  |  |  |  |  |  |  |
| Female | 0.983 | [0.966, 1.001] |  | 0.975 | [0.958, 0.993] | ** | - | - | - | - | - | - | 0.936 | [0.900, 0.974] | ** | 1.136 | [1.085, 1.190] | *** |
| Main work (Ref = Non-agricultural) |  |  |  |  |  |  |  |  |  |  |  |  |  |  |  |  |  |  |
| Economic inactivity | 1.069 | [1.032, 1.107] | *** | 1.050 | [1.014, 1.088] | ** | 1.159 | [1.087, 1.236] | *** | 0.993 | [0.909, 1.085] |  | 1.108 | [1.029, 1.194] | ** | 1.241 | [1.137, 1.356] | *** |
| Agricultural labor | 1.071 | [1.045, 1.097] | *** | 1.071 | [1.046, 1.097] | *** | 1.182 | [1.123, 1.245] | *** | 1.006 | [0.956, 1.059] |  | 1.125 | [1.070, 1.183] | *** | 1.213 | [1.148, 1.281] | *** |
| Hospitalization in adulthood (Ref = No) |  |  |  |  |  |  |  |  |  |  |  |  |  |  |  |  |  |  |
| At least once | 0.927 | [0.907, 0.949] | *** | 0.912 | [0.891, 0.932] | *** | 0.939 | [0.895, 0.985] | ** | 0.943 | [0.898, 0.991] | * | 0.906 | [0.864, 0.950] | *** | 0.843 | [0.799, 0.888] | *** |
| Birth Group (Ref = 1917-1948) |  |  |  |  |  |  |  |  |  |  |  |  |  |  |  |  |  |  |
| Birth cohort: 1949-1958 | 0.846 | [0.828, 0.864] | *** | 0.850 | [0.832, 0.869] | *** | 0.795 | [0.754, 0.837] | *** | 0.901 | [0.856, 0.948] | *** | 0.902 | [0.861, 0.945] | *** | 0.951 | [0.901, 1.003] |  |
| Birth cohort: 1959-1969 | 0.730 | [0.713, 0.747] | *** | 0.725 | [0.708, 0.743] | *** | 0.628 | [0.595, 0.662] | *** | 0.756 | [0.716, 0.799] | *** | 0.842 | [0.799, 0.887] | *** | 0.890 | [0.840, 0.944] | *** |
| Intercept | 74.130 | [71.529, 76.825] | *** | 79.559 | [76.738, 82.484] | *** | 100.096 | [92.485, 108.333] | *** | 107.541 | [98.245, 117.717] | *** | 112.009 | [103.321, 121.427] | *** | 108.658 | [99.196, 119.023] | *** |
| Log(scale) | 0.259 | [0.252, 0.266] | *** | 0.238 | [0.231, 0.246] | *** | 0.208 | [0.193, 0.224] | *** | 0.177 | [0.160, 0.197] | *** | 0.341 | [0.324, 0.359] | *** | 0.454 | [0.435, 0.473] | *** |
| AIC | 34573.4 | | | 29548.8 | | | 6545.9 | | | 3805.3  2,979 | | | 15975.9 | | | 20535.3 | | |
| Sample size | 5,654 | | | 5,850 | | | 3,128 | | |  |  |  | 6,072 | | | 5,980 | | |

*Note. * p<0.05; ** p<0.01; *** p<0.001. The results are derived from Accelerated Failure Time (AFT) Weibull models. TR is the Time ratio (exp(β)) from AFT models. The reported coefficients for Log(scale) are exp(log(scale)), representing the scales of AFT models.*

Figure A8. Forest plots for AFT model results

Table A11. AFT models for duration before the first health check-ups (Age <= 75)

|  | Blood Pressure Test | | | Blood Test | | | Breast Exam | | | Prostate Cancer Exam | | | Dental Exam | | | Vision Exam | | |
| --- | --- | --- | --- | --- | --- | --- | --- | --- | --- | --- | --- | --- | --- | --- | --- | --- | --- | --- |
| Predictors | TR | 95% CI |  | TR | 95% CI |  | TR | 95% CI |  | TR | 95% CI |  | TR | 95% CI |  | TR | 95% CI |  |
| Urban-rural (Ref = Urban residents with rural *hukou*) | | | | | | | | | | | | | | | | | | |
| Rural residents with rural *hukou* | 1.017 | [1.004, 1.031] | * | 1.031 | [1.017, 1.046] | *** | 0.997 | [0.970, 1.024] |  | 1.027 | [0.988, 1.067] |  | 1.068 | [1.036, 1.100] | *** | 1.089 | [1.052, 1.128] | *** |
| Urban *hukou* holder | 0.895 | [0.880, 0.911] | *** | 0.921 | [0.905, 0.937] | *** | 0.890 | [0.862, 0.918] | *** | 0.935 | [0.896, 0.977] | ** | 0.855 | [0.826, 0.886] | *** | 0.783 | [0.752, 0.814] | *** |
| Education (Ref = Illiterate) |  |  |  |  |  |  |  |  |  |  |  |  |  |  |  |  |  |  |
| Less than Middle school | 0.948 | [0.934, 0.962] | *** | 0.945 | [0.931, 0.960] | *** | 0.950 | [0.923, 0.979] | *** | 0.968 | [0.926, 1.012] |  | 0.908 | [0.879, 0.939] | *** | 0.843 | [0.810, 0.878] | *** |
| Middle school | 0.900 | [0.886, 0.914] | *** | 0.906 | [0.892, 0.921] | *** | 0.874 | [0.848, 0.901] | *** | 0.991 | [0.945, 1.040] |  | 0.871 | [0.841, 0.903] | *** | 0.736 | [0.705, 0.768] | *** |
| High school | 0.848 | [0.831, 0.865] | *** | 0.868 | [0.850, 0.885] | *** | 0.811 | [0.782, 0.841] | *** | 0.967 | [0.916, 1.020] |  | 0.783 | [0.751, 0.816] | *** | 0.586 | [0.558, 0.615] | *** |
| College and above | 0.689 | [0.667, 0.712] | *** | 0.713 | [0.691, 0.735] | *** | 0.640 | [0.607, 0.674] | *** | 0.844 | [0.790, 0.901] | *** | 0.639 | [0.602, 0.678] | *** | 0.418 | [0.391, 0.448] | *** |
| Marital (Ref = Continuously married) |  |  |  |  |  |  |  |  |  |  |  |  |  |  |  |  |  |  |
| Ever disrupted or never married | 1.014 | [0.999, 1.029] |  | 1.018 | [1.003, 1.034] | * | 1.049 | [1.019, 1.081] | ** | 1.019 | [0.977, 1.062] |  | 1.008 | [0.977, 1.040] |  | 0.973 | [0.938, 1.008] |  |
| Gender (ref = Male) |  |  |  |  |  |  |  |  |  |  |  |  |  |  |  |  |  |  |
| Female | 1.000 | [0.989, 1.012] |  | 0.981 | [0.970, 0.993] | ** | / | / | / | / | / | / | 0.950 | [0.928, 0.974] | *** | 1.200 | [1.167, 1.234] | *** |
| Main work (Ref = Non-agricultural) |  |  |  |  |  |  |  |  |  |  |  |  |  |  |  |  |  |  |
| Economic inactivity | 1.049 | [1.028, 1.071] | *** | 1.055 | [1.033, 1.078] | *** | 1.103 | [1.065, 1.141] | *** | 1.048 | [0.979, 1.123] |  | 1.072 | [1.026, 1.119] | ** | 1.166 | [1.109, 1.227] | *** |
| Agricultural labor | 1.039 | [1.024, 1.054] | *** | 1.052 | [1.037, 1.067] | *** | 1.104 | [1.075, 1.135] | *** | 1.008 | [0.976, 1.042] |  | 1.067 | [1.037, 1.098] | *** | 1.146 | [1.110, 1.183] | *** |
| Hospitalization in adulthood (Ref = No) |  |  |  |  |  |  |  |  |  |  |  |  |  |  |  |  |  |  |
| At least once | 0.930 | [0.916, 0.944] | *** | 0.895 | [0.881, 0.908] | *** | 0.950 | [0.921, 0.979] | ** | 0.930 | [0.899, 0.962] | *** | 0.905 | [0.878, 0.934] | *** | 0.874 | [0.844, 0.905] | *** |
| Birth Group (Ref = 1917-1948) |  |  |  |  |  |  |  |  |  |  |  |  |  |  |  |  |  |  |
| Birth cohort: 1949-1958 | 0.894 | [0.881, 0.906] | *** | 0.898 | [0.886, 0.911] | *** | 0.820 | [0.793, 0.847] | *** | 0.930 | [0.899, 0.961] | *** | 0.907 | [0.881, 0.934] | *** | 0.965 | [0.933, 0.997] | * |
| Birth cohort: 1959-1969 | 0.764 | [0.753, 0.776] | *** | 0.771 | [0.759, 0.783] | *** | 0.641 | [0.620, 0.662] | *** | 0.819 | [0.788, 0.851] | *** | 0.827 | [0.801, 0.854] | *** | 0.904 | [0.872, 0.937] | *** |
| Region (Ref = East) |  |  |  |  |  |  |  |  |  |  |  |  |  |  |  |  |  |  |
| Middle | 1.031 | [1.018, 1.045] | *** | 1.018 | [1.004, 1.031] | ** | 1.091 | [1.065, 1.117] | *** | 1.014 | [0.980, 1.049] |  | 1.102 | [1.072, 1.132] | *** | 1.117 | [1.083, 1.153] | *** |
| West | 1.044 | [1.031, 1.058] | *** | 1.023 | [1.010, 1.037] | *** | 1.067 | [1.041, 1.093] | *** | 0.994 | [0.961, 1.028] |  | 1.111 | [1.081, 1.143] | *** | 1.060 | [1.027, 1.094] | *** |
| Intercept | 68.268 | [66.701, 69.871] | *** | 75.249 | [73.473, 77.067] | *** | 100.633 | [95.898, 105.603] | *** | 106.006 | [99.092, 113.403] | *** | 107.838 | [102.469, 113.488] | *** | 98.456 | [92.931, 104.310] | *** |
| Log(scale) | 0.274 | [0.269, 0.279] | *** | 0.255 | [0.250, 0.260] | *** | 0.219 | [0.210, 0.228] | *** | 0.191 | [0.178, 0.204] | *** | 0.367 | [0.357, 0.378] | *** | 0.474 | [0.462, 0.485] | *** |
| AIC | 95758.4 | | | 83053.5 | | | 20521.6 | | | 9574.4  8,137 | | | 48499.9 | | | 58927.5 | | |
| Sample size | 15,084 | | | 15,836 | | | 8,542 | | |  |  |  | 16,543 | | | 16,220 | | |

*Note. * p<0.05; ** p<0.01; *** p<0.001. The results are derived from Accelerated Failure Time (AFT) Weibull models. TR is the Time ratio (exp(β)) from AFT models. The reported coefficients for Log(scale) are exp(log(scale)), representing the scales of AFT models.*

| Table A12. AFT Models for Duration Before the First Health Check-ups with Provincial Fixed Effects | | | | | | | | | | | | | | | | | | |
| --- | --- | --- | --- | --- | --- | --- | --- | --- | --- | --- | --- | --- | --- | --- | --- | --- | --- | --- |
|  | Blood Pressure Test | | | Blood Test | | | Breast Exam | | | Prostate Cancer Exam | | | Dental Exam | | | Vision Exam | | |
| Predictors | TR | 95% CI |  | TR | 95% CI |  | TR | 95% CI |  | TR | 95% CI |  | TR | 95% CI |  | TR | 95% CI |  |
| Urban-rural (Ref = Urban residents with rural *hukou*) | | | | | | | | | | | | | | | | | | |
| Rural residents with rural *hukou* | 1.017 | [1.003, 1.031] | * | 1.026 | [1.012, 1.041] | *** | 1.012 | [0.984, 1.041] |  | 1.013 | [0.975, 1.051] |  | 1.067 | [1.035, 1.100] | *** | 1.089 | [1.052, 1.128] | *** |
| Urban *hukou* holder | 0.896 | [0.881, 0.911] | *** | 0.914 | [0.898, 0.929] | *** | 0.904 | [0.876, 0.933] | *** | 0.933 | [0.895, 0.973] | ** | 0.849 | [0.820, 0.880] | *** | 0.781 | [0.751, 0.811] | *** |
| Education (Ref = Illiterate) |  |  |  |  |  |  |  |  |  |  |  |  |  |  |  |  |  |  |
| Less than Middle school | 0.934 | [0.921, 0.947] | *** | 0.933 | [0.920, 0.947] | *** | 0.947 | [0.920, 0.975] | *** | 0.947 | [0.910, 0.985] | ** | 0.895 | [0.867, 0.924] | *** | 0.836 | [0.805, 0.868] | *** |
| Middle school | 0.896 | [0.883, 0.910] | *** | 0.898 | [0.883, 0.912] | *** | 0.880 | [0.854, 0.906] | *** | 0.954 | [0.913, 0.997] | * | 0.858 | [0.829, 0.889] | *** | 0.731 | [0.702, 0.761] | *** |
| High school | 0.842 | [0.826, 0.859] | *** | 0.858 | [0.841, 0.875] | *** | 0.813 | [0.784, 0.843] | *** | 0.942 | [0.896, 0.991] | * | 0.777 | [0.746, 0.810] | *** | 0.585 | [0.559, 0.613] | *** |
| College and above | 0.716 | [0.693, 0.739] | *** | 0.722 | [0.700, 0.745] | *** | 0.655 | [0.621, 0.689] | *** | 0.833 | [0.783, 0.885] | *** | 0.650 | [0.613, 0.689] | *** | 0.428 | [0.402, 0.457] | *** |
| Marital (Ref = Continuously married) |  |  |  |  |  |  |  |  |  |  |  |  |  |  |  |  |  |  |
| Ever disrupted or never married | 1.028 | [1.014, 1.042] | *** | 1.039 | [1.025, 1.054] | *** | 1.072 | [1.042, 1.103] | *** | 1.040 | [1.003, 1.078] | * | 1.041 | [1.010, 1.072] | ** | 0.990 | [0.958, 1.023] |  |
| Gender (ref = Male) |  |  |  |  |  |  |  |  |  |  |  |  |  |  |  |  |  |  |
| Female | 0.997 | [0.986, 1.008] |  | 0.979 | [0.968, 0.990] | *** | / | / | / | / | / | / | 0.950 | [0.928, 0.972] | *** | 1.174 | [1.143, 1.206] | *** |
| Main work (Ref = Non-agricultural) |  |  |  |  |  |  |  |  |  |  |  |  |  |  |  |  |  |  |
| Economic inactivity | 1.064 | [1.043, 1.085] | *** | 1.067 | [1.046, 1.089] | *** | 1.110 | [1.072, 1.149] | *** | 1.053 | [0.991, 1.119] |  | 1.103 | [1.057, 1.150] | *** | 1.164 | [1.110, 1.220] | *** |
| Agricultural labor | 1.039 | [1.024, 1.053] | *** | 1.050 | [1.036, 1.065] | *** | 1.090 | [1.060, 1.120] | *** | 1.006 | [0.976, 1.037] |  | 1.057 | [1.028, 1.087] | *** | 1.130 | [1.096, 1.165] | *** |
| Hospitalization in adulthood (Ref = No) |  |  |  |  |  |  |  |  |  |  |  |  |  |  |  |  |  |  |
| At least once | 0.928 | [0.915, 0.942] | *** | 0.895 | [0.883, 0.908] | *** | 0.944 | [0.917, 0.973] | *** | 0.933 | [0.905, 0.962] | *** | 0.912 | [0.886, 0.940] | *** | 0.880 | [0.852, 0.910] | *** |
| Birth Group (Ref = 1917-1948) |  |  |  |  |  |  |  |  |  |  |  |  |  |  |  |  |  |  |
| Birth cohort: 1949-1958 | 0.847 | [0.836, 0.858] | *** | 0.850 | [0.839, 0.862] | *** | 0.779 | [0.755, 0.803] | *** | 0.897 | [0.869, 0.925] | *** | 0.859 | [0.836, 0.883] | *** | 0.899 | [0.872, 0.928] | *** |
| Birth cohort: 1959-1969 | 0.726 | [0.715, 0.736] | *** | 0.731 | [0.720, 0.741] | *** | 0.605 | [0.586, 0.624] | *** | 0.790 | [0.762, 0.820] | *** | 0.785 | [0.761, 0.810] | *** | 0.843 | [0.814, 0.872] | *** |
| Province (Ref = Beijing) |  |  |  |  |  |  |  |  |  |  |  |  |  |  |  |  |  |  |
| Shanghai | 1.016 | [0.912, 1.131] |  | 1.114 | [1.004, 1.236] | * | 0.920 | [0.805, 1.052] |  | 1.035 | [0.854, 1.255] |  | 1.014 | [0.855, 1.202] |  | 0.822 | [0.678, 0.997] | * |
| Yunnan | 1.226 | [1.131, 1.328] | *** | 1.210 | [1.127, 1.299] | *** | 1.187 | [1.072, 1.314] | ** | 1.067 | [0.937, 1.216] |  | 1.366 | [1.200, 1.554] | *** | 1.199 | [1.035, 1.390] | * |
| Neimenggu | 1.070 | [0.988, 1.158] |  | 1.138 | [1.061, 1.221] | *** | 1.202 | [1.087, 1.328] | *** | 1.169 | [1.019, 1.341] | * | 1.225 | [1.081, 1.389] | ** | 1.221 | [1.055, 1.414] | ** |
| Jilin | 1.150 | [1.059, 1.250] | *** | 1.241 | [1.151, 1.339] | *** | 1.280 | [1.146, 1.431] | *** | 1.137 | [0.983, 1.315] |  | 1.220 | [1.069, 1.393] | ** | 1.133 | [0.974, 1.319] |  |
| Sichuan | 1.114 | [1.030, 1.205] | ** | 1.119 | [1.044, 1.199] | ** | 1.203 | [1.090, 1.329] | *** | 1.107 | [0.974, 1.258] |  | 1.282 | [1.133, 1.450] | *** | 1.184 | [1.027, 1.366] | * |
| Tianjin | 1.069 | [0.966, 1.182] |  | 1.194 | [1.089, 1.309] | *** | 1.069 | [0.937, 1.220] |  | 1.207 | [0.967, 1.506] |  | 1.224 | [1.027, 1.459] | * | 1.072 | [0.886, 1.296] |  |
| Anhui | 1.106 | [1.021, 1.198] | * | 1.170 | [1.090, 1.257] | *** | 1.283 | [1.149, 1.433] | *** | 1.101 | [0.963, 1.260] |  | 1.319 | [1.158, 1.503] | *** | 1.284 | [1.105, 1.493] | ** |
| Shandong | 1.063 | [0.983, 1.150] |  | 1.109 | [1.035, 1.188] | ** | 1.132 | [1.028, 1.246] | * | 1.170 | [1.028, 1.333] | * | 1.174 | [1.039, 1.326] | ** | 1.060 | [0.921, 1.221] |  |
| Shanxi(山西) | 1.136 | [1.046, 1.234] | ** | 1.220 | [1.132, 1.314] | *** | 1.387 | [1.226, 1.570] | *** | 1.281 | [1.090, 1.506] | ** | 1.276 | [1.114, 1.461] | *** | 1.277 | [1.091, 1.494] | ** |
| Guangdong | 1.187 | [1.095, 1.287] | *** | 1.197 | [1.114, 1.286] | *** | 1.215 | [1.095, 1.348] | *** | 1.116 | [0.975, 1.278] |  | 1.085 | [0.957, 1.229] |  | 1.154 | [0.996, 1.337] |  |
| Guangxi | 1.153 | [1.062, 1.251] | *** | 1.164 | [1.082, 1.252] | *** | 1.136 | [1.024, 1.259] | * | 1.093 | [0.954, 1.252] |  | 1.210 | [1.063, 1.379] | ** | 1.137 | [0.978, 1.322] |  |
| Xinjiang | 1.105 | [1.004, 1.216] | * | 1.081 | [0.992, 1.178] |  | 1.199 | [1.038, 1.384] | * | 1.066 | [0.893, 1.274] |  | 1.137 | [0.967, 1.336] |  | 1.182 | [0.985, 1.419] |  |
| Jiangsu | 1.107 | [1.022, 1.199] | * | 1.119 | [1.043, 1.201] | ** | 1.125 | [1.019, 1.243] | * | 1.068 | [0.937, 1.218] |  | 1.204 | [1.061, 1.366] | ** | 1.102 | [0.953, 1.274] |  |
| Jiangxi | 1.175 | [1.085, 1.272] | *** | 1.191 | [1.110, 1.277] | *** | 1.219 | [1.103, 1.347] | *** | 1.114 | [0.978, 1.268] |  | 1.321 | [1.165, 1.498] | *** | 1.226 | [1.061, 1.416] | ** |
| Heibei | 1.065 | [0.983, 1.154] |  | 1.201 | [1.118, 1.290] | *** | 1.072 | [0.972, 1.183] |  | 1.126 | [0.983, 1.291] |  | 1.242 | [1.094, 1.410] | *** | 1.182 | [1.020, 1.369] | * |
| Heinan | 1.076 | [0.994, 1.164] |  | 1.114 | [1.039, 1.193] | ** | 1.159 | [1.051, 1.278] | ** | 1.077 | [0.947, 1.224] |  | 1.171 | [1.036, 1.324] | * | 1.157 | [1.003, 1.335] | * |
| Zhejiang | 1.030 | [0.951, 1.116] |  | 1.016 | [0.947, 1.090] |  | 1.030 | [0.934, 1.136] |  | 1.064 | [0.932, 1.215] |  | 0.985 | [0.870, 1.115] |  | 0.982 | [0.850, 1.136] |  |
| Hubei | 1.119 | [1.032, 1.213] | ** | 1.093 | [1.018, 1.175] | * | 1.194 | [1.073, 1.329] | ** | 1.185 | [1.027, 1.367] | * | 1.207 | [1.060, 1.374] | ** | 1.221 | [1.049, 1.421] | * |
| Hunan | 1.168 | [1.078, 1.267] | *** | 1.139 | [1.061, 1.223] | *** | 1.162 | [1.050, 1.286] | ** | 1.119 | [0.979, 1.279] |  | 1.353 | [1.188, 1.541] | *** | 1.153 | [0.995, 1.337] |  |
| Gansu | 1.159 | [1.066, 1.259] | *** | 1.228 | [1.138, 1.324] | *** | 1.339 | [1.187, 1.511] | *** | 1.081 | [0.940, 1.244] |  | 1.262 | [1.102, 1.444] | *** | 1.206 | [1.030, 1.412] | * |
| Fujian | 1.227 | [1.128, 1.336] | *** | 1.217 | [1.128, 1.314] | *** | 1.302 | [1.150, 1.474] | *** | 1.125 | [0.969, 1.307] |  | 1.065 | [0.932, 1.217] |  | 1.201 | [1.022, 1.410] | * |
| Guizhou | 1.376 | [1.242, 1.523] | *** | 1.306 | [1.191, 1.433] | *** | 1.365 | [1.132, 1.647] | ** | 1.168 | [0.962, 1.417] |  | 1.270 | [1.071, 1.506] | ** | 1.064 | [0.884, 1.280] |  |
| Liaoning | 1.138 | [1.049, 1.234] | ** | 1.269 | [1.178, 1.366] | *** | 1.118 | [1.009, 1.238] | * | 1.151 | [0.997, 1.329] |  | 1.227 | [1.077, 1.398] | ** | 1.148 | [0.988, 1.336] |  |
| Chongqing | 1.159 | [1.063, 1.265] | *** | 1.191 | [1.100, 1.291] | *** | 1.169 | [1.030, 1.327] | * | 1.002 | [0.866, 1.160] |  | 1.285 | [1.102, 1.498] | ** | 1.051 | [0.889, 1.242] |  |
| Shanxi(陕西) | 1.096 | [1.010, 1.189] | * | 1.168 | [1.086, 1.256] | *** | 1.079 | [0.975, 1.194] |  | 1.120 | [0.976, 1.285] |  | 1.082 | [0.953, 1.229] |  | 0.981 | [0.846, 1.137] |  |
| Qinghai | 1.132 | [1.030, 1.244] | * | 1.164 | [1.064, 1.273] | *** | 1.146 | [0.988, 1.329] |  | 1.029 | [0.854, 1.239] |  | 1.270 | [1.054, 1.531] | * | 1.035 | [0.853, 1.257] |  |
| Heilongjiang | 1.170 | [1.076, 1.272] | *** | 1.231 | [1.142, 1.328] | *** | 1.191 | [1.071, 1.326] | ** | 1.075 | [0.934, 1.237] |  | 1.189 | [1.042, 1.357] | * | 1.390 | [1.188, 1.626] | *** |
| Intercept | 66.021 | [60.987, 71.470] | *** | 70.378 | [65.596, 75.509] | *** | 94.699 | [85.815, 104.502] | *** | 101.687 | [89.138, 116.003] | *** | 101.516 | [89.547, 115.085] | *** | 97.944 | [84.750, 113.192] | *** |
| Log(scale) | 0.274 | [0.269, 0.278] | *** | 0.255 | [0.250, 0.260] | *** | 0.220 | [0.211, 0.229] | *** | 0.189 | [0.177, 0.201] | *** | 0.368 | [0.358, 0.379] | *** | 0.468 | [0.457, 0.479] | *** |
| AIC | 104611.43 | | | 90839.99 | | | 21680.95 | | | 11083.38  8,898 | | | 52904.40 | | | 64867.72 | | |
| Sample size | 16,374 | | | 17,214 | | | 9,307 | | |  |  |  | 18,032 | | | 17,724 | | |

*Note. * p<0.05; ** p<0.01; *** p<0.001. The results are derived from Accelerated Failure Time (AFT) Weibull models. TR is the Time ratio (exp(β)) from AFT models. The reported coefficients for Log(scale) are exp(log(scale)), representing the scales of AFT models.*

Table A13. AFT Models for Duration Before the First Health Check-ups (Gender = Male)

|  | Blood Pressure Test | | | Blood Test | | | Dental Exam | | | Vision Exam | | |
| --- | --- | --- | --- | --- | --- | --- | --- | --- | --- | --- | --- | --- |
| Predictors | TR | 95% CI |  | TR | 95% CI |  | TR | 95% CI |  | TR | 95% CI |  |
| Urban-rural (Ref = Urban residents with rural *hukou*) |  |  |  |  |  |  |  |  |  |  |  |  |
| Rural residents with rural *hukou* | 1.014 | [0.991, 1.039] |  | 1.033 | [1.009, 1.059] | ** | 1.082 | [1.028, 1.139] | ** | 1.088 | [1.031, 1.149] | ** |
| Urban *hukou* holder | 0.862 | [0.838, 0.887] | *** | 0.878 | [0.853, 0.904] | *** | 0.822 | [0.776, 0.870] | *** | 0.735 | [0.691, 0.780] | *** |
| Education (Ref = Illiterate) |  |  |  |  |  |  |  |  |  |  |  |  |
| Less than Middle school | 0.919 | [0.894, 0.945] | *** | 0.922 | [0.895, 0.949] | *** | 0.828 | [0.775, 0.884] | *** | 0.763 | [0.710, 0.820] | *** |
| Middle school | 0.856 | [0.831, 0.881] | *** | 0.872 | [0.846, 0.899] | *** | 0.790 | [0.737, 0.846] | *** | 0.633 | [0.588, 0.682] | *** |
| High school | 0.793 | [0.767, 0.820] | *** | 0.836 | [0.808, 0.866] | *** | 0.692 | [0.643, 0.746] | *** | 0.489 | [0.451, 0.530] | *** |
| College and above | 0.653 | [0.621, 0.687] | *** | 0.685 | [0.652, 0.719] | *** | 0.579 | [0.525, 0.638] | *** | 0.356 | [0.321, 0.395] | *** |
| Marital (Ref = Continuously married) |  |  |  |  |  |  |  |  |  |  |  |  |
| Ever disrupted or never married | 1.038 | [1.012, 1.065] | ** | 1.035 | [1.008, 1.062] | ** | 1.040 | [0.985, 1.099] |  | 1.014 | [0.958, 1.074] |  |
| Main work (Ref = Non-agricultural) |  |  |  |  |  |  |  |  |  |  |  |  |
| Economic inactivity | 1.072 | [1.031, 1.116] | *** | 1.079 | [1.036, 1.123] | *** | 1.170 | [1.070, 1.279] | *** | 1.212 | [1.109, 1.325] | *** |
| Agricultural labor | 1.041 | [1.019, 1.063] | *** | 1.049 | [1.027, 1.071] | *** | 1.062 | [1.017, 1.109] | ** | 1.133 | [1.084, 1.185] | *** |
| Hospitalization in adulthood (Ref = No) |  |  |  |  |  |  |  |  |  |  |  |  |
| At least once | 0.911 | [0.890, 0.933] | *** | 0.874 | [0.854, 0.895] | *** | 0.881 | [0.841, 0.924] | *** | 0.859 | [0.816, 0.903] | *** |
| Birth Group (Ref = 1917-1948) |  |  |  |  |  |  |  |  |  |  |  |  |
| Birth cohort: 1949-1958 | 0.847 | [0.828, 0.866] | *** | 0.865 | [0.846, 0.885] | *** | 0.877 | [0.837, 0.918] | *** | 0.886 | [0.843, 0.931] | *** |
| Birth cohort: 1959-1969 | 0.739 | [0.722, 0.758] | *** | 0.741 | [0.723, 0.759] | *** | 0.836 | [0.793, 0.880] | *** | 0.818 | [0.776, 0.863] | *** |
| Region (Ref = East) |  |  |  |  |  |  |  |  |  |  |  |  |
| Middle | 1.020 | [0.999, 1.043] |  | 0.997 | [0.976, 1.019] |  | 1.096 | [1.048, 1.146] | *** | 1.089 | [1.039, 1.143] | *** |
| West | 1.045 | [1.022, 1.068] | *** | 1.018 | [0.996, 1.041] |  | 1.104 | [1.055, 1.157] | *** | 1.047 | [0.997, 1.098] |  |
| Intercept | 75.574 | [72.743, 78.515] | *** | 84.285 | [81.016, 87.685] | *** | 134.480 | [123.276, 146.702] | *** | 135.750 | [123.768, 148.892] | *** |
| Log(scale) | 0.338 | [0.331, 0.346] | *** | 0.313 | [0.305, 0.321] | *** | 0.447 | [0.430, 0.465] | *** | 0.580 | [0.563, 0.598] | *** |
| AIC | 52700.41 | | | 46068.86 | | | 26786.34 | | | 37637.76 | | |
| Sample size | 7,936 | | | 8,357 | | | 8,769 | | | 8,560 | | |
| *Note. * p<0.05; ** p<0.01; *** p<0.001. The results are derived from Accelerated Failure Time (AFT) Weibull models. TR is the Time ratio (exp(β)) from AFT models. The reported coefficients for Log(scale) are exp(log(scale)), representing the scales of AFT models.* | | | | | | | | | | | | |

Table A14. AFT Models for Duration Before the First Health Check-ups (Gender = Female)

|  | Blood Pressure Test | | | Blood Test | | | Dental Exam | | | Vision Exam | | |
| --- | --- | --- | --- | --- | --- | --- | --- | --- | --- | --- | --- | --- |
| Predictors | TR | 95% CI |  | TR | 95% CI |  | TR | 95% CI |  | TR | 95% CI |  |
| Urban-rural (Ref = Urban residents with rural *hukou*) |  |  |  |  |  |  |  |  |  |  |  |  |
| Rural residents with rural *hukou* | 1.009 | [0.995, 1.023] |  | 1.021 | [1.006, 1.035] | ** | 1.046 | [1.013, 1.080] | ** | 1.058 | [1.025, 1.091] | *** |
| Urban *hukou* holder | 0.922 | [0.906, 0.939] | *** | 0.957 | [0.939, 0.975] | *** | 0.879 | [0.846, 0.914] | *** | 0.861 | [0.830, 0.893] | *** |
| Education (Ref = Illiterate) |  |  |  |  |  |  |  |  |  |  |  |  |
| Less than Middle school | 0.937 | [0.924, 0.950] | *** | 0.938 | [0.924, 0.952] | *** | 0.928 | [0.899, 0.959] | *** | 0.887 | [0.859, 0.915] | *** |
| Middle school | 0.912 | [0.898, 0.928] | *** | 0.910 | [0.895, 0.926] | *** | 0.893 | [0.860, 0.927] | *** | 0.832 | [0.802, 0.863] | *** |
| High school | 0.873 | [0.854, 0.893] | *** | 0.874 | [0.854, 0.895] | *** | 0.828 | [0.790, 0.867] | *** | 0.718 | [0.687, 0.751] | *** |
| College and above | 0.760 | [0.730, 0.792] | *** | 0.769 | [0.739, 0.801] | *** | 0.675 | [0.629, 0.725] | *** | 0.536 | [0.501, 0.575] | *** |
| Marital (Ref = Continuously married) |  |  |  |  |  |  |  |  |  |  |  |  |
| Ever disrupted or never married | 1.027 | [1.013, 1.042] | *** | 1.045 | [1.030, 1.061] | *** | 1.032 | [1.001, 1.065] | * | 1.007 | [0.978, 1.037] |  |
| Main work (Ref = Non-agricultural) |  |  |  |  |  |  |  |  |  |  |  |  |
| Economic inactivity | 1.049 | [1.027, 1.071] | *** | 1.060 | [1.037, 1.083] | *** | 1.071 | [1.026, 1.118] | ** | 1.126 | [1.081, 1.173] | *** |
| Agricultural labor | 1.037 | [1.019, 1.056] | *** | 1.054 | [1.035, 1.073] | *** | 1.070 | [1.033, 1.109] | *** | 1.123 | [1.086, 1.162] | *** |
| Hospitalization in adulthood (Ref = No) |  |  |  |  |  |  |  |  |  |  |  |  |
| At least once | 0.951 | [0.935, 0.967] | *** | 0.916 | [0.900, 0.931] | *** | 0.943 | [0.910, 0.978] | ** | 0.924 | [0.893, 0.955] | *** |
| Birth Group (Ref = 1917-1948) |  |  |  |  |  |  |  |  |  |  |  |  |
| Birth cohort: 1949-1958 | 0.849 | [0.837, 0.862] | *** | 0.847 | [0.834, 0.860] | *** | 0.842 | [0.816, 0.870] | *** | 0.910 | [0.883, 0.938] | *** |
| Birth cohort: 1959-1969 | 0.718 | [0.707, 0.729] | *** | 0.730 | [0.718, 0.743] | *** | 0.745 | [0.719, 0.772] | *** | 0.846 | [0.817, 0.875] | *** |
| Region (Ref = East) |  |  |  |  |  |  |  |  |  |  |  |  |
| Middle | 1.032 | [1.018, 1.046] | *** | 1.027 | [1.013, 1.042] | *** | 1.096 | [1.064, 1.129] | *** | 1.100 | [1.069, 1.132] | *** |
| West | 1.028 | [1.014, 1.042] | *** | 1.014 | [1.000, 1.028] | * | 1.075 | [1.044, 1.107] | *** | 1.035 | [1.006, 1.065] | * |
| Intercept | 71.131 | [69.461, 72.841] | *** | 75.201 | [73.380, 77.067] | *** | 99.391 | [94.365, 104.686] | *** | 92.991 | [88.605, 97.593] | *** |
| Log(scale) | 0.212 | [0.207, 0.216] | *** | 0.200 | [0.195, 0.205] | *** | 0.292 | [0.280, 0.303] | *** | 0.289 | [0.278, 0.300] | *** |
| AIC | 51398.95 | | | 44506.99 | | | 26006.19 | | | 26513.30 | | |
| Sample size | 8,438 | | | 8,857 | | | 9,263 | | | 9,164 | | |
| *Note. * p<0.05; ** p<0.01; *** p<0.001. The results are derived from Accelerated Failure Time (AFT) Weibull models. TR is the Time ratio (exp(β)) from AFT models. The reported coefficients for Log(scale) are exp(log(scale)), representing the scales of AFT models.* | | | | | | | | | | | | |
